# Bioinspired helical-artificial fibrous muscle structured tubular soft actuators

**DOI:** 10.1126/sciadv.adh3350

**Published:** 2023-06-23

**Authors:** Zhiming Hu, Yanlin Zhang, Hanqing Jiang, Jiu-an Lv

**Affiliations:** ^1^ Key Laboratory of 3D Micro/Nano Fabrication and Characterization of Zhejiang Province, School of Engineering, Westlake University, Hangzhou 310024, Zhejiang, China.; ^2^ School of Engineering, Westlake University, Hangzhou 310030, Zhejiang, China.; ^3^ Westlake Institute for Advanced Study, Hangzhou 310024, Zhejiang, China.; ^4^ Research Center for Industries of the Future, Westlake University, Hangzhou 310030, Zhejiang, China.

## Abstract

Biological tubular actuators show diverse deformations, which allow for sophisticated deformations with well-defined degrees of freedom (DOF). Nonetheless, synthetic active tubular soft actuators largely only exhibit few simple deformations with limited and undesignable DOF. Inspired by 3D fibrous architectures of tubular muscular hydrostats, we devised conceptually new helical-artificial fibrous muscle structured tubular soft actuators (HAFMS-TSAs) with locally tunable molecular orientations, materials, mechanics, and actuation via a modular fabrication platform using a programmable filament winding technique. Unprecedentedly, HAFMS-TSAs can be endowed with 11 different morphing modes through programmable regulation of their 3D helical fibrous architectures. We demonstrate a single “living” artificial plant rationally structured by HAFMS-TSAs exhibiting diverse photoresponsive behaviors that enable adaptive omnidirectional reorientation of its hierarchical 3D structures in the response to environmental irradiation, resembling morphing intelligence of living plants in reacting to changing environments. Our methodology would be significantly beneficial for developing sophisticated soft actuators with designable and tunable DOF.

## Teaser

Helical-artificial-fibrous-muscle structured tubular soft actuators enable designable and tunable multimodal deformations.

## INTRODUCTION

Creating tubular soft actuators that exhibit diverse controllable and programmable shape transformations in response to environmental stimuli would enable broad scientific and engineering applications (*1*–*4*). However, synthetic tubular actuators using soft active materials (SAMs) largely only exhibit few simple deformations (linear contraction/expansion) with limited and undesignable degrees of freedom (DOFs), arising from their spatially monotonic molecular orientation, material, mechanics, and actuation. In recent years, there has been growing interest to leverage liquid crystal elastomers (LCEs) to devise smart active devices (e.g., actuators and pumps) in the scientific community (*5*, *6*) because these appealing SAMs can not only provide large-scale, reversible deformations but also impressively enable encoding “morphing instructions” into their own materials and architectures via patterning LC alignments and thus allow for the creation of compact, programmable, small-scale morphing devices/robots, which would offer potential technological applications extremely difficult to address with rigid systems that need many auxiliary components (tether, values, etc.) (*7*). Nonetheless, achieving programmable deformations in LCEs has proved extremely challenging, especially for three-dimensional (3D) hierarchical structures because it requires both precisely programmed geometric shapes and well-controlled LC alignments (*8*–*10*). Conventional preparation techniques for LCEs are limited to only fabricate thin, flat 2D films with either uniform or nonuniform LC alignment induced by mechanical stretching, rubbed surfaces, or applied magnetic field (*11*–*13*). To prepare 3D tubular LCE structures, fabrication techniques that combine molding with mechanical stretching or rubbed alignment layer have been developed (*14*, *15*). However, these traditional molding-based approaches can only gain simple, uniform LC orientation architecture along their long axis, which inevitably leads to single and monotonous morphing behavior (linear contraction/expansion). Therefore, unconventional tubular LCE actuators with tunable 3D geometries and spatial molecular orientation architectures, which can be constructed through a readily accessible, highly efficient, flexible fabrication platform, are urgently needed for full exploitation of the potential of LCEs as compact smart active devices/robots with diverse morphing modes and sophisticated deformations.

**Fig. 1.**
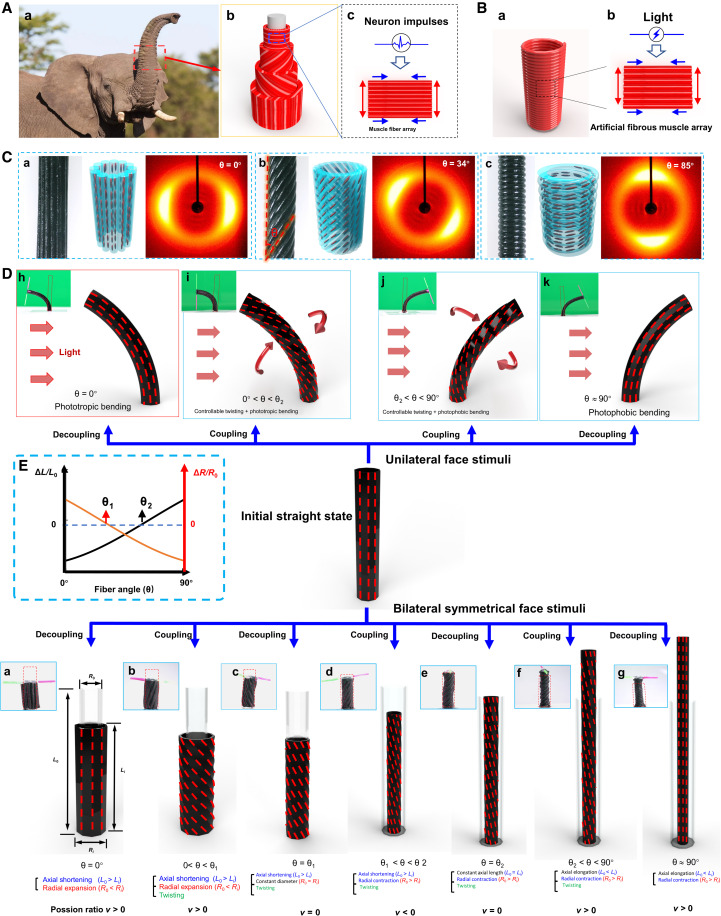
Design principle inspired by 3D fibrous architectures of biological tubular actuatorsABCDE. (**A**) Muscular fiber architecture and driving principle of the elephant trunk (one kind of muscular hydrostats). Anisotropic deformation of the muscle fiber array is induced by neuron impulses. Photo credit: Shutterstock. (**B**) Bioinspired tubular artificial muscle composed of directionally arranged microfiber actuator arrays. Anisotropic contraction of the microfiber actuator array is driven by light. (**C**) Tunable 3D helical structures of HAFMS-TSAs. Photographs showing the morphologies, 3D spatial alignment of LCs, 2D wide-angle x-ray diffraction (WXRD) patterns for HAFMS-TSAs with θ = 0°, 34°, and 85°, respectively. (**D**) Eleven morphing modes shown by HAFMS-TSAs. The dashed red lines marked on the surfaces of HAFMS-TSAs are used as reference lines to indicate the different deformations of HAFMS-TSAs. (**E**) Schematics showing the two boundary values of critical fiber winding angles (θ_1_ and θ_2_). Δ*L* and Δ*R* indicate the length change and the diameter change of deformed HAFMS-TSA. *L*_0_ and *R*_0_ denote the initial length and the initial diameter of the HAFMS-TSA, respectively.

Looking into natural tubular actuators for inspiration in addressing this challenge, we noticed that the 3D fibrous architectures of trunks of elephants, the entirely soft tubular actuators without the assistance of bones and joints featuring a closely packed 3D array of muscle fibers can well define their DOF (well known as muscle hydrostats; Fig. 1A) (*16*), which enables remarkably diverse, complex, and highly controlled shape transformations (*17*, *18*). For elephant trunks, muscle fibers are directionally arranged and winded around the trunk’s long axis to form the tubular muscle layer (Fig. 1A, b). Multiple muscle layers with varied orientation directions together constitute the 3D tubular muscle hydrostat. Assignment of specific alignment direction of muscle fibers for directional muscle layers grants the ability to yield diverse morphing modes that include not only single-deformation modes, such as shortening, elongation, bending, and torsion, but also their compound morphing modes coupling two or more deformations (*16*). These biological tubular actuators can gain multimodal and programmable shape transformations through directionally arranging responsive muscle fiber into 3D architectures. If we were able to leverage artificial muscle fibers as basic building blocks and directionally and spatially arrange them (Fig. 1B), then a straightforward and effective method to construct 3D tubular soft actuators with diverse morphing modes and designable DOF would be achieved.

Inspired by thnd programmed e 3D fibrous architectures of the biological tubular actuators, we developed a programmable filament winding platform for bottom-up construction of helical-artificial fibrous muscle structured tubular soft actuators (HAFMS-TSAs) that exhibit diverse tunable morphing behaviors embedded ain their bioinspired 3D helical fiber architectures (Fig. 1C). Unlike previous tubular actuators fabricated by active soft materials that only show few conventional deformations (mainly axial shortening/elongation + radial expansion/contraction, with positive Poisson’s ratio ν > 0), our HAFMS-TSAs having on-demand 3D LC director fields and geometries, as well as locally tunable materials, LC order, mechanics, and actuations, unprecedentedly not only exhibit up to 11 different morphing modes (Fig. 1D) but also enable generating unconventional deformations with unusual Poisson’s ratio ν (ν = 0 or ν < 0) (Fig. 1D, c, f, or d), which have yet been achieved by synthetic active 3D soft materials. In addition, two critical boundary values (θ_1_ and θ_2_, 0 < θ_1_ < θ_2_ < 90°) of winding angle [θ, which is indicated in Fig. 1C (b)] have been first discovered (when θ of a HAFMS-TSA equals θ_1_ or θ_2_, the HAFMS-TSA can maintain its constant radial or axial length, respectively, meanwhile generating complex deformations) and leveraged to achieve the decoupling and coupling among axial deformation (elongating or shortening), radial deformation (contraction or expansion), out-plane deformation (directional bending), and torsional deformation (twisting) in the 3D actuators. This discovery offers an effective and versatile approach to well-define rich DOFs and thus access sophisticated manipulation of morphing behaviors, e.g., photophobic, phototropic, and photonastic omnidirectional morphing behaviors simultaneously and inherently generated in an intelligent artificial plant to allow for adaptive, autonomous transformations of its hierarchical 3D morphing structures.

## RESULTS

Thanks to two critical boundary values of fiber winding angles θ_1_ and θ_2_ unprecedentedly discovered in this work (Fig. 1E), HAFMS-TSAs can generate diverse controllable, complex shape transformations, which are shown in Fig. 1D. When the winding angle θ takes different values, specifically θ = 0°, 0° < θ < θ_1_, θ = θ_1_, θ_1_ < θ < θ_2,_ θ = θ_2_, θ_2_ < θ < 90°, or θ = 90°, upon bilateral symmetrical face stimuli, seven morphing modes can be induced respectively: (a) axial shortening + radial expansion, (b) twisting + axial shortening + radial expansion, (c) axial shortening + twisting while maintaining constant diameter, (d) axial shortening + radial contraction + twisting, (e) twisting + radial contraction while maintaining constant axial length, (f) axial elongation + radial contraction + twisting, and (g) axial elongation + radial contraction (fig. S1A and movie S1). Upon unilateral face stimuli, when θ = 0°, 0° < θ < θ_2_, θ_2_ < θ < 90°, or θ = 90°, four morphing modes can be controllably generated: (h) phototropic bending, (i) phototropic bending coupled with controllable twisting, (j) photophobic bending coupled with controllable twisting, and (k) photophobic bending (fig. S1B and movie S2). It is highly impressive that the coupling and decoupling between distinct deformations, such as radial deformation and twisting (when θ = θ_1_, decoupled; when θ ≠ θ_1_ and 0° < θ < 90°, coupled), axial deformation and twisting (when θ = θ_2_, decoupled; when θ ≠ θ_2_ and 0° < θ < 90°, coupled), phototropic bending and twisting (when θ = 0°, decoupled; when 0° < θ < θ_2_, coupled), and photophobic bending and twisting (when θ = 90°, decoupled; when θ_2_ < θ < 90°, coupled) can be well defined in HAFMS-TSAs through regulation of the fiber winding angles. In addition, morphing modes (i) and (j) combine directional bending with twisting motion, featuring high DOF. Moreover, unlike existing active soft actuators that largely show positive Poisson’s ratios, morphing modes (c), (d), and (e) of HAFMS-TSAs show zero or negative Poisson’s ratios, remarkable shape-changing characteristics of metamaterials. These five unconventional morphing modes [(c), (d), (e), (i), and (j)] have yet not been realized by the state-of-the-art synthetic active soft materials.

**Fig. 2.**
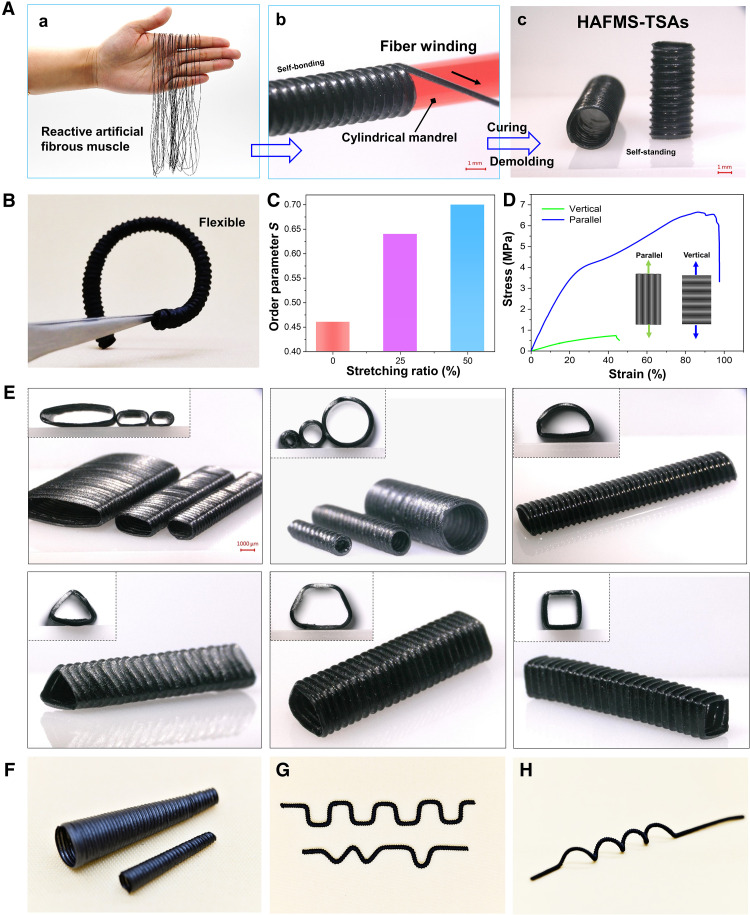
Tunable 3D geometries for HAFMS-TSAs. (**A**) Experimental photographs exhibiting the bioinspired modular fabrication of HAFMS-TSAs through programmable filament winding technique. (**B**) Flexible, curved HAFMS-TSA. (**C**) Order parameters of fibrous building units can be regulated by the stretching ratio. (**D**) Uniaxial tensile testing results for the wall of a HAFMS-TSA along the fiber orientation direction and vertical to the fiber orientation direction, respectively. (**E**) HAFMS-TSAs with well-defined geometries. (**F**) Asymmetrical HAFMS-TSAs with tapered shapes. (**G**) Regular and irregular S-shaped HAFMS-TSAs. (**H**) A coil spring–shaped HAFMS-TSA.

To straightforwardly mimic directional fibrous architectures and thus realize the design concept underlying the actuation principle of the natural tubular actuators, we devise a bioinspired modular fabrication methodology that is composed of two stages. In stage 1, we used a screw mold or a tubular mold to shape LCE oligomers into reactive fibrous precursor in which a chemical cross-link reaction is still going on over time (fig. S2; see details in Materials and Methods); this reactive fiber in which thiol-functionalized cross-linkers dispersed in acrylate-functional LCE oligomers would be used as the building block for the following construction of various HAFMS-TSAs. In stage 2, the freshly prepared fibrous precursor is mechanically stretched and directionally, closely winded onto a designed mandrel with target 3D geometry. The mechanical stretching can not only induce LC alignment in the fiber but also allow for regulation of LC order (Fig. 2C), while the winding angle of the stretched fiber is used to dictate 3D helical director field of the winded tubular actuator. Keep winding the fiber until the mandrel’s surface is fully covered by the fiber array. The winded fiber arrays can exactly replicate 3D geometric shape of the used mandrel (Fig. 2A, b). The thiol-functional groups and acrylate groups left on the fibers’ surface offer the chemical anchors for adjoining fibers in contact, enabling self-bonding of the contacted fibers through the thiol-acrylate click reaction and thereby ensuring the mechanical robustness in interface strength without the need for any artificial participation and operations (e.g., gluing; Fig. 2, B and D). After the chemical bonding reaction is completed, self-standing, mechanically robust HAFMS-TSAs are thus obtained after the removal of the mandrel (Fig. 2A, c). This programmable filament winding platform has the capabilities to not only enable 3D helix of director fields (Fig. 1C and fig. S3) but also arbitrarily tune geometric shapes of fabricated tubular actuators, for example, rectangle, triangle, rhombus, trapezoid, etc. (Fig. 2E), allowing for the fabrication of asymmetric HAFMS-TSAs with various tapered shapes and even more complex hierarchical 3D shapes (e.g., S-shaped, helical shape, etc.; Fig. 2, F to H, and fig. S4), which is extremely difficult if not impossible to achieve by previously reported fabrication methods for tubular soft actuators (*19*, *20*). In our approach, artificial fibrous muscles with sufficient mechanical strength provide the building blocks that can be easily handled, displaced, and shaped during fabrication. With the use of shape-tunable mandrels as the 3D shaping templates, any desired 3D shapes can be easily built. Control of the fiber winding angle offers a feasible and readily available way to regulate the 3D orientation profile of LC mesogens in the constructed 3D actuators. Our methodology offers a high DOF approach for the design and fabrication of complex 3D actuators.

**Fig. 3.**
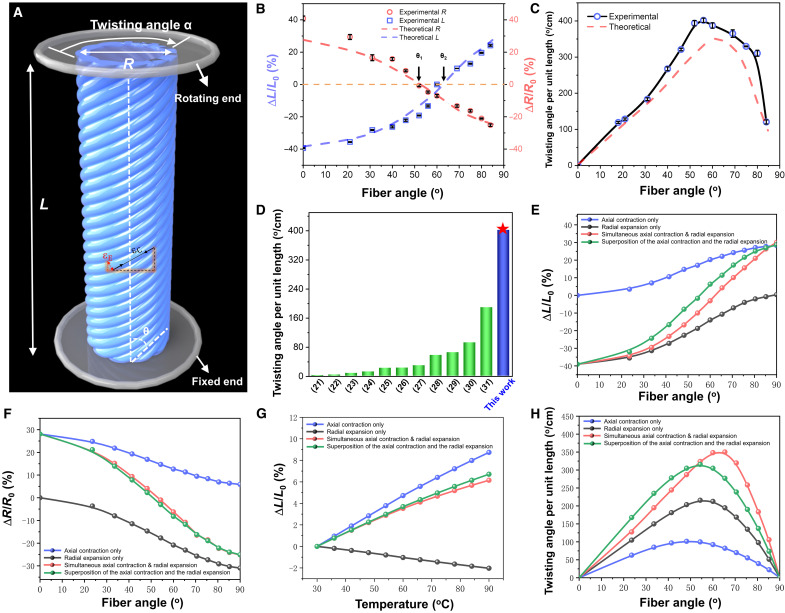
Programmable actuation of HAFMS-TSAsABCDEFGH. (**A**) Geometry and infinite model of the HAFMS-TSA. α and θ indicate the twisting angle and fiber angle, respectively. (**B**) Model-predicted and experimentally measured length change ratio (Δ*L*/*L*_0_) and diameter change ratio (Δ*R*/*R*_0_) as a function of θ. (**C**) Twisting angle per unit length of HAFMS-TSA as a function of θ. The inner diameter and length of the tubular actuator are ~2 and ~10 mm, respectively. The diameter of the fiber is ~0.7 mm. (**D**) Statistics graph showing twist angle (α) per unit length for previous reports and this work. (**E**) Model-predicted length change ratio (Δ*L*/*L*_0_) as a function of θ under axial contraction only (blue curve), radial expansion only (gray curve), simultaneous axial contraction and radial expansion (red curve), and superposition of the axial contraction and the radial expansion (green curve), respectively. (**F**) Model-predicted diameter change ratio (Δ*R*/*R*_0_) as a function of θ under axial contraction only (blue curve), radial expansion only (gray curve), simultaneous axial contraction and radial expansion (red curve), and superposition of the axial contraction and the radial expansion (green curve), respectively. (**G**) Model-predicted length change ratio (Δ*L*/*L*_0_) as a function of temperature under axial contraction only (blue curve), radial expansion only (gray curve), simultaneous axial contraction and radial expansion (red curve), and superposition of the axial contraction and the radial expansion (green curve), respectively. (**H**) Model-predicted twisting angle per unit length as a function of θ under axial contraction only (blue curve), radial expansion only (gray curve), simultaneous axial contraction and radial expansion (red curve), and superposition of the axial contraction and the radial expansion (green curve), respectively.

Understanding the effect of the fiber winding angle on the achievable morphing behaviors of HAFMS-TSAs is crucial for our design, which would allow for controllable actuation and sophisticated operations in soft robotic applications. The deformation behavior of HAFMS-TSAs arises from the anisotropic deformation of the structural units of helically arranged active fibers, which can be regarded as the superposition effect of the deformations of the helically arranged fiber units. The fiber units contract in their longitudinal direction and expand in their radial direction. Note that, similar to the important biomechanical feature of muscle fiber of muscular hydrostat (*16*), the volume of the fibrous building blocks remains constant when deformed (fig. S5), which means any decrease in one dimension will simultaneously lead to a compensatory increase in at least one other dimension. Upon light exposure or, equivalently, temperature increase, the contraction strain (ε*_C_*) along the fiber direction can be decomposed into two orthogonal components, with one along the axial direction of a HAFMS-TSA and the other along the circumferential direction (Fig. 3A). Similarly, the radial expansion strain (ε*_E_*) of the fiber unit can also be decomposed into two orthogonal components (Fig. 3A). Thus, the overall strain (ε*_L_*) of the deformation in the axial direction of a HAFMS-TSA can be described as the superposition of the corresponding components of the contraction strain and the expansion strain of the fiber unit, which can be described as ε*_L_* = ε*_E_* sin θ + ε*_c_* cos θ, while the overall circumferential strain (ε*_p_*) can be denoted as ε*_p_* = ε*_E_* cos θ + ε*_c_* sin θ.

Unprecedentedly, two boundary values of critical fiber winding angles (θ_1_ and θ_2_) were discovered. When the winding angle θ is equal to θ = θ_1_ or θ = θ_2_, the circumferential strain ε*_p_* and the axial strain ε*_L_* would be equal to 0, which means that the HAFMS-TSA with θ = θ_1_ can maintain their diameter constant while the HAFMS-TSA with θ = θ_2_ can keep their length constant during stimuli-responsive deformations. Plotting ε*_L_* and ε*_p_* as a function of θ, the variation of morphing modes can be observed with the change of fiber winding angle. As shown in Fig. 3B, when 0 < θ < θ_1_, HAFMS-TSA contracts along its long axis and expands in its circumferential direction (ν > 0) and simultaneously generates twisting deformation. When θ is close to or equal to θ_1_, it is interesting to observe that ν = 0; the HAFMS-TSA can keep its perimeter constant while producing shortening and twisting motion simultaneously. When θ_1_ < θ < θ_2_, the HAFMS-TSA begins to contract in both circumferential direction and longitudinal direction (ν < 0), showing unusual shape change distinguished from the previously reported common soft actuators that contract in the longitudinal direction while also generating compensatory expansion in the width direction (ν > 0). When θ is close to or equal to θ_2_, the longitudinal length of the HAFMS-TSA can remain unchanged, and HAFMS-TSA simultaneously generates twisting motion combined with contraction in its circumferential direction. When θ_2_ < θ < 90°, the HAFMS-TSA elongates along their longitudinal direction while contracting in their circumferential direction (ν > 0). Through the critical fiber winding angles (0°, θ_1_, θ_2_, and 90°), the decoupling and coupling of diverse deformations can be achieved. When θ = 0° or 90°, the decoupling of twisting from the axial and radial deformations can be achieved. When θ = θ_1_ and θ = θ_2_, the decoupling of the radial deformation from twisting and axial deformation and the decoupling of axial deformation from twisting and radial deformation can be realized. In the angular interval divided by these two critical fiber winding angles (0 < θ < θ_1_, θ_1_ < θ < θ_2_, and θ_2_ < θ < 90°), the coupling among twisting, axial deformation, radial deformation can be gained. To figure out the influence of θ on the twisting angles of HAFMS-TSAs, we measured the twisting angle per unit length (α) of HAFMS-TSAs with varied θ. As shown in Fig. 3C, α first increases and then decreases as θ increases. When θ = 56°, the HAFMS-TSA shows the maximum α of 401°/cm, which is twice higher than the highest record of twisting angle of existing tubular soft actuators (~189°/cm; Fig. 3D) (*21*–*31*). Regulation of fiber winding angles can not only control the direction of deformations (when 0 < θ < θ_2_, shortening; when θ_2_ < θ < 90°, elongating) but also enable precise control of the magnitude of the twisting deformations. All these suggest that we can make use of tunable 3D helical-fiber structures to precisely define morphing behaviors with controllable DOF to gain diverse sophisticated shape transformations, which is in agreement with the design principle underlying the programmable actuation of the biological tubular actuators with 3D fibrous architectures.

The deformation mechanism to achieve rich morphing modes is the combined effect of the intrinsic mechanical properties of the deformable fiber units and the winding angle θ. Specifically, upon light exposure, photoactive fibrous units exhibit transversely isotropic behavior, i.e., shortening in the axial direction and expanding isotropically in the lateral direction. Combined with the winding angle θ that defines the 3D helical director field (Fig. 1C), the hierarchically structured tubular actuators exhibit rich deformation modes. It is now straightforward to understand the extreme modes that all fibrous muscles are along the axial direction (θ = 0° or model 1) or the circumferential direction (θ = 90°, mode 7). For helically structured tubular actuators (0° < θ < 90°), two critical winding angles θ_1_ and θ_2_ would emerge. At the θ_2_, the projection of the shortening along the fiber direction and the expanding along the circumferential direction of the fiber cancels out and thus leads to an overall constant axial length and completely pure twisting due to its critical 3D helical architecture (i.e., θ = θ_2_ or mode 5), while the other critical winding angle θ_1_ (<θ_2_) defines a vanishing Poisson’s ratio, at which the lateral shrinkage due to the shortening effect along the fiber direction and the lateral expansion perpendicular to the fiber direction balance out.

To quantitatively understand this mechanism and offer a theoretical tool to precisely predict and visualize the morphing behaviors of HAFMS-TSAs, we have conducted numerical calculations using Fung orthotropic material in ABAQUS, in which an analogy between the light-induced deformation and thermal strain was established, the thermal expansion along the axial direction was adopted to mimic the axial deformation of the fiber upon light exposure, its coefficient (<0 for shortening) was obtained by fitting the experimental data, and the coefficient of its lateral counterpart (>0) was calculated by assuming constant volume before and after light exposure. Namely, the fiber was modeled as a material with transversely isotropic thermal expansion. Details can be found in Materials and Methods. Figure 3E shows that with just the axial shortening effect, no matter the winding angle θ, the tubular actuator always exhibits shortening mode; while with just the lateral expansion effect, the opposite trend is observed (Fig. 3F), and their combination provides a range of axial deformation, from shortening to expansion depending on the θ. It can be observed that, here, the individually superposed deformation (green curve in Fig. 3G) has an apparent deviation from the combined one (red curve in Fig. 3G), which can be explained by the large deformation effect. A similar trend is also presented for the twisting angle (Fig. 3H). All these simulation results suggest that the rich morphing behaviors of HAFMS-TSAs arise from the coupling among the fiber’s axial contraction and lateral expansion and its 3D helically orientated structure, which provide the theoretical toolset to help precisely tailor the morphing behaviors that emerged in HAFMS-TSAs.

**Fig. 4.**
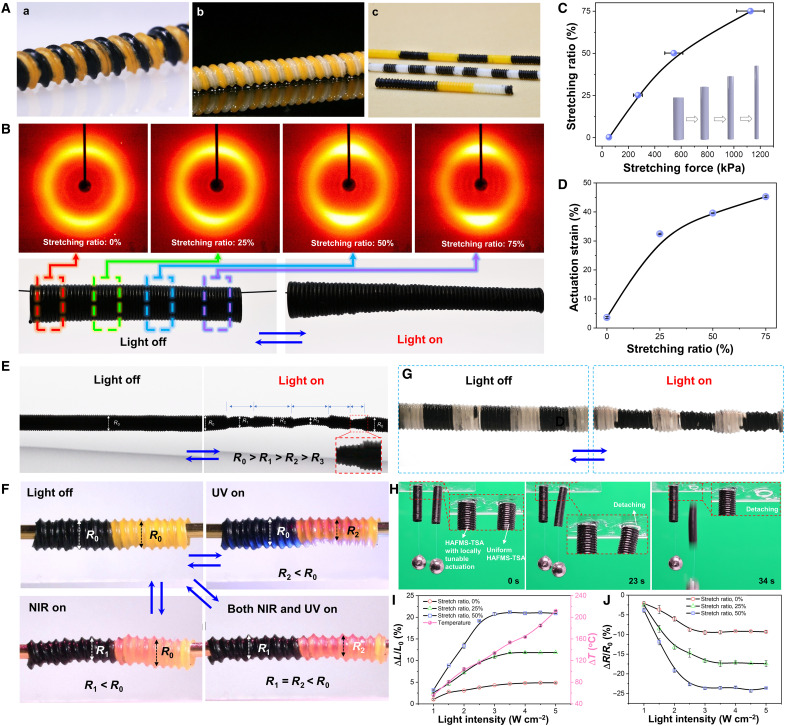
Locally tunable materials, LC order, and actuations. (**A**) Programmable materials in the single HAFMS-TSA. (**B**) Graded LC order enables a HAFMS-TSA to transform from a symmetric (cylindrical) shape into an asymmetric (tapered) shape when exposed to light irradiation. 2D-WXRD patterns at different parts of the HAFMS-TSA indicate varying LC order along its longitudinal direction. (**C**) Quantified stretching ratio as a function of the applied stretching force. (**D**) Actuation strain as a function of stretching ratio. (**E**) Locally varied LC orders allow controllable generation of five regions with different shape changes in a single HAFMS-TSA. *R*_0_ is the initial diameter of the HAFMS-TSA. *R*_1_, *R*_2_, and *R*_3_ are the diameters for deformed parts 1, 2, 3, and 4, respectively. Part 5 is deformed into a tapered shape. (**F**) A material-programmed HAFMS-TSA made of NIR- and UV-responsive material shows locally tunable photodeformation. Under the irradiation of either NIR or UV light, the local change in the diameter of the composited actuator can be induced, while the overall change of the diameter can be generated upon the simultaneous irradiation of NIR and UV light. (**G**) A material-programmed HAFMS-TSA composed of alternating non-photoresponsive and photoresponsive LCE materials reversibly transformed between a cylinder structure and a pearl necklace–like structure by light. (**H**) Proof-of-concept demonstration of preventing stress concentration near the interface between morphing HAFMS-TSAs and other connected elements through locally tunable actuation strain/stress. Experimental photographs show that the HAFMS-TSA with uniform actuation strain/stress, glued on a glass substrate, detaching from a glass substrate upon light irradiation, whereas the HAFMS-TSA with locally reduced actuation strain/stress still stayed attached to the substrate. (**I**) Length change ratios of HAFMS-TSA (θ = 85°; inner diameter, 1 mm) when irradiated by light with different intensities. (**J**) Curves showing diameter change ratio of HAFMS-TSA (θ = 85°; inner diameter, 1 mm) as a function of light intensity.

During the fabrication of HAFMS-TSAs, the spontaneous and robust chemical self-bonding between multimaterials and structures offers an effective and versatile approach for seamlessly building 3D soft actuators with local tunability of materials, LC order, mechanics, and actuations. The chemical self-bonding reaction can occur not only between parts of the fiber stretched with varying stretching ratios but also between different material formulations (Fig. 4A). As shown in Fig. 4B, by gradually increasing the stretching force on the fiber during the winding process, we can gradually increase the stretching ratio of the fiber (Fig. 4, C and D) so that the fiber at different positions in the same single tubular actuator can have varied LC order. As shown in Fig. 4B, the LC order can gradually increase along the HAFMS-TSA’s long axis via a graded increase of the stretching ratio of the used fiber. Such LC-order-gradient HAFMS-TSA can reversibly switch between symmetrical tubular shape and asymmetrical tapered shape by light (movie 3, part 1). By controllably changing the stretching ratio of the fibers during the winding process, we can gain on-demand diameter change at any desired part of the single actuator. As shown in Fig. 4E, we can achieve three distinct diameter changes at three different locations within a single HAFMS-TSA and even simultaneously make an asymmetric change, forming a tapered shape at the right end of the same HAFMS-TSA (movie S3, part 2). Leveraging our programmable platform, different LCE materials can be introduced into the desired parts of a single HAFMS-TSA, thus realizing the spatial programming of the materials (Fig. 4, F and G). As shown in Fig. 4F, a multimaterial HAFMS-TSA, half made of near-infrared (NIR)–responsive LCE fiber and the other half composed of ultraviolet (UV)–responsive LCE fiber, allows for multiwavelength and local response (movie S3, part 3). All these graded deformations and local deformations are difficult to realize by conventional tubular soft actuators. Moreover, we can locally tune the actuation strain of a HAFMS-TSA to effectively avoid stress concentration–induced detaching [Fig. 4H and movie S3 (part 5)], which is critically important for tubular soft actuators when they need to partly bond with other nonresponsive rigid materials or elements to integrate into actuating devices or robotic systems. Our versatile filament winding platform enables highly efficient fabrication of 3D actuators with the unique merit of locally tunable and programmable actuations, which would be useful for a wide range of engineering applications that need controllable modulation of 3D geometries (*32*, *33*).

Note that previous passive-actuation tubular actuators/robots relied on strain limiters (rigid parts) to induce anisotropic deformation, not only leading to laborious multiple-step fabrications but also requiring cable systems or elastic inflatable structures for driving shape changes (*21*, *26*). As a result, many auxiliary components, such as tethers, valves, connecter, batteries, microprocessors, and pumps (or motors), must be involved to gain actuation and control, which make the robotic system bulky and complicated and thereby difficult to design and fabricate compact, integrated, miniaturized soft robotic system. Whereas our platform shows the capabilities not only to use a single type of entirely soft active LCE materials to achieve the fabrication of tubular soft actuators, offering a feasible solution to overcome a formidable challenge in the materials science community (*34*, *35*), but also to enable embedding morphological information inside the material through the helical fiber architecture. The morphing instruction within the material itself via spatial directional-arranged fiber architectures not only largely reduces the required number of active components necessary for complex shape morphing but also saves much time and energy that would otherwise be consumed in signal processing and feedback for morphing control, sharply simplifying fabrication, driving, and control of soft robotic systems (*36*, *37*).

**Fig. 5.**
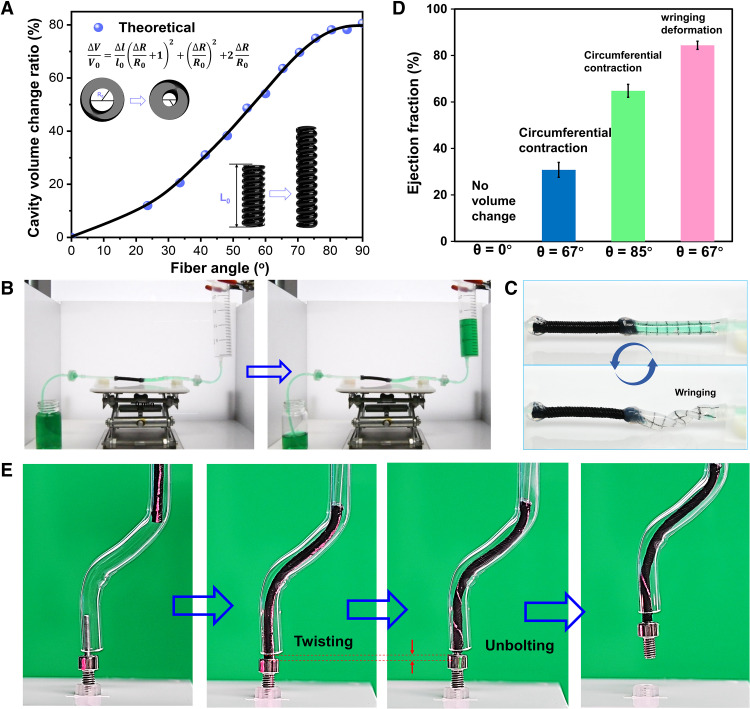
Versatile functionalities of HAFMS-TSAs. (**A**) Predicted cavity volume change ratio of HAFMS-TSAs as a function of θ. (**B**) Photographs showing fluid pumping driven by leveraging the cavity volume change of HAFMS-TSA upon light irradiation. (**C**) Wringing deformation is driven by the twisting motion of the HAFMS-TSA, and it causes torsional buckling of the elastic tube, leading to its collapse and squeezing the most fluid out of the wrung tube. (**D**) Large ejection fraction achieved by wringing motion, which is induced by the twisting motion of HAFMS-TSAs. (**E**) HAFMS-TSA adapt to a meandering pipe to unscrew a bolt through a photo-driven twisting motion.

We further demonstrate that our HAFMS-TSAs can be engineered for three interesting applications, namely, efficiently pumping fluids, small-scale soft robotic tentacles, and ILAPs. Soft, active pumps that respond to environmental stimuli and produce large-volume change offer considerable promise in microfluidics, soft robotics, and wearable pumping devices. Tubular LCEs that can effectively convert environmental stimuli into large-scale, reversible shape changes are highly promising for those engineering applications. However, previously reported tubular LCEs with simple orientation along their long axis cannot be used to pump fluids because there is no volume change in their cavity when undergoing longitudinal contraction upon external stimuli (*19*), which can be verified by both experimental results and theoretical simulation (Fig. 5, A and D). The lack of change in volume implies that previously reported tubular LCEs cannot exert mechanical pressure on the fluids enclosed in their cavities, thus failing to effectively pump fluids. As a result, the previously reported tubular LCEs have been seldom demonstrated as active pumps and mainly used as soft actuators for driving motions (*20*, *38*). Thanks to 3D helical fibrous architectures, our HAFMS-TSAs exhibit remarkable changes in volume that allow for effective pumping functionalities (Fig. 5A). The experimental setup using the concept of helical fiber–structured tubular LCEs as a reciprocating micropump is shown in Fig. 5B (fig. S6 and movie S4). We have offered important new insights and crucial design points of the usage of helical active fiber architectures to achieve effective pumping functions. In our soft pumping system, ejection fraction (EF), the volumetric fraction of fluid ejected from the cavity of a tubular soft actuator for each deformation cycle, is used to evaluate pumping efficiency. For our HAFMS-TSAs, the increase of θ can enhance EF, and the maximum EF can reach 65% when θ = 85°.

To enhance ejection fraction to improve mechanical proficiency, we further devised a powerful soft pump that exhibits a higher ejection fraction by leveraging the unusual, high-DOF deformation of mammal’s hearts, that is, the wringing motion. For mammal hearts, the length change of myocardial fiber is 15 to 20% at most. If the blood ejection simply results from the contraction of myocardial fibers, then the EF would be 15 to 20%, whereas the actual EF is three times this value, up to 60 to 70% (*39*). The high EF arises from the involvement of the wringing motion. The helical cardiac design of muscle fibers enables the wringing motion of the mammal’s heart, which allows the natural 15% muscle fiber shortening to generate the unusually high EF (*40*). Inspired by this biological principle, we created a conceptually new soft pump that makes use of the twisting motion of our HAFMS-TSA to wring an elastomer tube connected with the twisting HAFMS-TSA to achieve a high ejection fraction via the torsional buckling of the wrung tube. As shown in Fig. 5C, our twisting pump can twist, collapse, and extensively squeeze the fluid to achieve a high EF up to 84%. This bioinspired pumping strategy based on wringing motion can be extended to other SAM systems to improve pumping efficiency (fig. S7 and movie S5). Moreover, our HAFMS-TSA can serve as a small-scale soft robotic tentacle that is able to not only grab a bolt through photo-driven radical contraction and unscrew it in a straight state [fig. S8A and movie S6 (part 1)] but also exert torque to a bolt in its curved states [fig. S8B and movie S6 (part 2)], even adapt to a meandering pipe to unscrew a bolt through photo-driven twisting motion although the multiple parts of the tentacle are curved [Fig. 5E and movie S6 (part 3)], which implies great potential in applications of high-DOF micromechanical operations and miniature soft manipulator arms (*41*).

In addition, we demonstrate that our HAFMS-TSAs can be developed to construct ILAPs that are able to act/behave adaptively and automatically in response to environmental stimuli. Plants are intelligent biological material systems, which enable distinct adaptive and dynamical movements for their different organs in response to varying solar radiation conditions, allowing for self-regulating 3D hierarchical architectures. Plants’ photoresponsive movements fall into three categories: (i) phototropic movements that plant organs orient toward the light, (ii) photophobic movements that plant organs orient away from the light, and (iii) photonastic movements that plant organs morph and orient upon light irradiation independent of the direction of the light. Different organs (e.g., stem, branch, and petiole) in a plant have varied categories of photoinduced plant movements, thus generating diverse spatial orientations of responding plant organs and leading to adaptive 3D hierarchical architectures. However, existing manmade intelligent material systems largely show photonastic movements and seldom exhibit phototropic movements (*42*). No synthetic active soft material system can intrinsically respond and exhibit all three categories of light-induced movements shown by real living plants. Rationally leveraging our programmable HAFMS-TSA with tunable helical fibrous architectures, we designed and created an ILAP that displays all three categories of photoresponse movements, which not only enables ILAP reversible transition between a compact closed 2D state and 3D hierarchical opening state but also adaptively changing its 3D hierarchical structures according to light irradiation conditions.

**Fig. 6.**
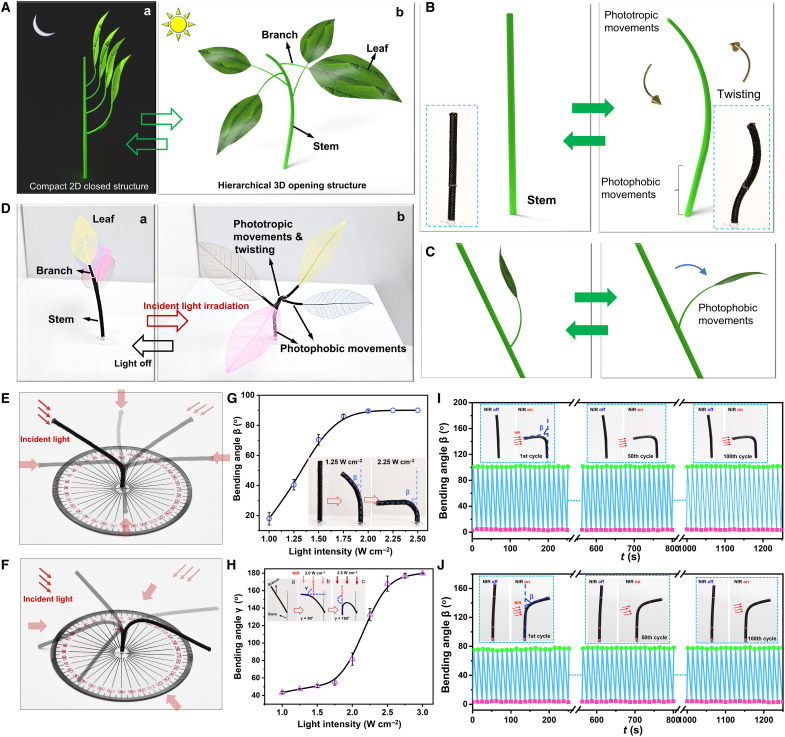
ILAPs constructed by HAFMS-TSAs. (**A**) Schematics showing the concept of an intelligent artificial plant. (**B**) Photo-driven movements of the stem. The stem uses a Janus structure composed of two different parts. The lower part (length, 15 mm) is made of the HAFMS-TSA with θ = 85° to enable photophobic movements, and the upper part (length, 40 mm) consists of HAFMS-TSA with θ = 40° to produce phototropic movements and large photonastic twisting. (**C**) Phototropic movements of the branch. (**D**) Proof-of-concept demonstration of the intelligent artificial plant. With varied light-driven orientation movements for different organs, the plant can adaptatively change its 3D hierarchical structures according to light irradiation conditions (e.g., illuminating direction and light intensity). (**E** and **F**) Photographs showing phototropic movements and photophobic movements enable the tunable and controllable 3D spatial reorientation of a HAFMS-TSA. (**G** and **H**) Light intensity–regulated directional bending can be used to tune the reorientation directions. The diagrams (G) and (H) show the bending angles of a HAFMS-TSA (θ = 40°; length, 40 mm; inner diameter, 2 mm) and a HAFMS-TSA (θ = 83°; length, 20 mm; inner diameter, 1 mm) upon different light intensities, respectively. The red arrows indicate the illuminating direction of the light. (**I** and **J**) One hundred cycles of phototropic bending of the HAFMS-TSA (θ = 0°) and photophobic bending of the HAFMS-TSA (θ = 83°) without obvious fatigue.

ILAP contains three types of organs: stem, branches, and leaves, which were designed to show distinct photo-reorientation movements (Fig. 6A). The stem uses a Janus structure composed of two different parts. As shown in Fig. 6B, the lower part (length, 15 mm; inner diameter, 2 mm) is made of the HAFMS-TSA with θ = 85° to enable photophobic movements, and the upper part (length, 40 mm; inner diameter, 2 mm) consists of the HAFMS-TSA with θ = 40° to produce both phototropic movements and large photonastic twisting. The photophobic bending of the lower part ensures that the center of gravity of the entire plant falls on the stem when the upper part is bending forward, providing good mechanical support during shape transformations and preventing the opening structure of ILAP from tipping over. Meanwhile, for the upper part, phototropic movements can drive ILAP facing toward the incident light, and simultaneously, strong twisting motion can make leaf distribution transition from the linear arrangement to 3D spatial arrangements, opening to gain a 3D hierarchical structure. The branches are made of the HAFMS-TSA with a large θ of 85° (length, 15 mm; inner diameter, 1 mm), displaying photophobic movements and relatively weak photonastic twisting, which enable them to reorient away from the light and thus increasing the angle between the branches and the stem (Fig. 6C), together with photonastic twisting of the branches, leading the leaves to reorient and make their blades face the incident light. Without light irradiation, ILAP exhibits a compact closed 2D structure, in which the stem stands upright, while the branches and leaves are arranged in a linear distribution on one lateral side of the stem, near a 2D planar distribution [Fig. 6, A (a) and D (a)]. Upon light irradiation, different organs of ILAP show varied categories of photo-reorientation movements (photophobic, phototropic, and photonastic) and reorient along diverse directions to yield adaptive 3D opening hierarchical structures [Fig. 6, A (b) and D (b), and movie S8]. Note that photo-reorientation movements are not only related to the incident direction of light (Fig. 6, E and F; figs. S9 to S11, and movie S7) but also regulated by the intensity of light (Fig. 6, G and H). For photoresponsive “organs,” the increase in light intensity leads to the increase of *γ* (the included angle *γ* between the initial orientation direction and the reoriented direction). When the light intensity reaches a critical value of 2 W/cm^2^, the photophobic branches would be reoriented to be perpendicular to the incident direction of the light (inset image b of Fig. 6H), realizing the maximum light interception. When the light intensity exceeds the threshold value, the photophobic organs will be gradually oriented parallel to the incident direction of the light to minimize light interception through the built-in feedback loop that inherently exists in the photomechanical properties of the material. Light intensity–regulated photophobic motion behaviors provide an effective self-protection mechanism for ILAP. Upon ambient and proper light irradiation, ILAP is fully opened to form adaptive 3D structures with the spatial distribution of leaves, which results from the omnidirectional phototropic movements of the stem, the omnidirectional photophobic movements of the branches, and the photonastic movements of the stem and branches, enabling each leaf’s blade perpendicular to the incident direction of light (facing the light) and the maximum light interception rate (fig. S12). When the light irradiation becomes stronger, the photophobic movements of the branches drive the leaves to generate large photophobic movements, reducing the exposure area of the leaves. When the light intensity reaches a threshold, the branches and leaves will be reoriented away from the light and parallel to the incident direction of the light (inset image c of Fig. 6H), minimizing their exposure area and effectively reducing the radiation damage, which much resembles the self-protection intelligence of automatically avoiding damage from high doses of solar radiation shown by real plants in nature (*43*). Moreover, we tested the deformation durability of a phototropic HAFMS-TSA and a photophobic HAFMS-TSA, respectively. As shown in Fig. 6 (I and J), the HAFMS-TSAs allow for lasting more than 100 bending cycles without obvious fatigue, demonstrating high durability that is critically important for real engineering applications. Our ILAP capable of adaptive and autonomous 3D morphing structures in response to varying radiation environments would be of great significance for the development of many cutting-edge technologies, such as enhanced artificial photosynthesis and solar harvest, solar sails for space stations and spaceships, self-regulating optical devices, etc.

## DISCUSSION

We have developed conceptually new artificial fibrous muscle structured tubular SAMs featured with freely encoded 3D tubular geometries and 3D helical molecular orientation, in which LC order, material formulation, mechanical properties, and actuations can be locally and spatially modulated in a single 3D structured actuator, allowing for programmable photo-triggered heterogeneous material responses and diverse shape transformations that are not achievable through traditional approaches. Our current report concentrates on the introduction of the basic principle; the potential of our methodology leveraging fibrous artificial-muscle placement–based strategy has yet to be fully explored. For example, if the automated fiber placement technique is used, then arbitrary 3D artificial muscle structures with well-defined spatial LC orientation profiles and high resolution in local LC order, materials, and mechanics can be manufactured. In addition, our HAFMS-TSAs demonstrate unique and counterintuitive behaviors, not only unprecedentedly exhibiting metamaterial-like deformations with unusual Poisson ratio ν = 0 or ν < 0 but also enabling coupling and decoupling of various different deformations, offering the versatile approaches to precisely tune complex shape transformations of the 3D actuators. We also provide new insight into leveraging the two boundary values of critical fiber winding angles (θ_1_ and θ_2_) to regulate complex morphing behaviors. With guidance from theoretical calculating and numerical modeling that help unravel the relationship between spatial molecular orientation profiles and corresponding shape-morphing behaviors, complex 3D-to-3D shape transformations/shifting/switching can be blueprinted, enabling larger freedom in actuation and functionality design. In addition, ILAP with designable DOF enables adaptive and autonomous 3D morphing intelligence, which have been previously largely only observed in living biomaterials, now achieved in synthetic materials. This “life-like” material that intrinsically shows the self-regulated morphing intelligence would be significantly beneficial for pushing technology boundaries of decentralized artificial intelligence for soft robotic systems. Our artificial fibrous muscle structured SAM system with high freedom in the design and programmable, adaptive 3D shape-morphing structures offers a myriad of possibilities to exploit controllable multimodal compact actuators for broad emerging technologies, such as interactive soft robots, soft pumps, artificial muscles, micro-mechanical systems, and beyond.

## MATERIALS AND METHODS

### Materials

LC monomer {1,4-bis-[4-(6-acryloyloxyhexyloxy)benzoyloxy]-2-methylbenzene; RM82} and photoresponsive azobenzene monomer {4,4′-bis[9-(acryloyloxy) propoxy]azobenzene; DA3AB} were purchased from Shijiazhuang Yesheng Chemical Technology Co. Ltd. and Beijing Realchem Technology Co. Ltd., respectively. The chain extender [3,6-dioxa-1,8-octanedithiol (DODT)], cross-linker [pentaerythritol tetrakis (3-mercaptopropionate) (PETMP)], catalyst [dipropylamine (DPA)], and NIR dopant (graphene) were obtained from TCI.

### Characterizations

NIR light (808 nm) was generated by laser sources (MDL-H-808-5W or FC-W808-50W, Changchun New Industries Optoelectronics Technology Co. Ltd.). The laser intensity was monitored by a laser power meter (TP100, Changchun New Industries Optoelectronics Technology Co. Ltd.). UV light was obtained from a light-emitting diode lamp (Omron ZUV-C30H for λ = 365 nm). The intensity of UV light was measured by a Newport 1917-R optical power meter with HIOKI 2018 photodetector. 2D x-ray diffraction patterns were obtained by Bruker D8 Venture diffractometer. The tensile stress-strain measurements of the wall of the HAFMS-TSA were performed using an Instron universal testing system (model 5943) at a tensile testing rate of 10 mm/min in air. Supplementary movies were recorded by a superresolution digital microscope (Keyence, VHX-1000C) or a digital camera [Canon, EOS 80D(W)].

### Preparation of the fibrous precursor

To gain the fibrous precursors, a screw mold or a tubular mold was used to shape LCE oligomers into the reactive fibers. First, a mixture formulated with 1.67:1 molar ratio of RM82 and DODT and 3:1 molar ratio of DODT:PETMP and 2 wt % graphene was dissolved in chloroform. Thiol groups and acrylate groups were equimolar. After ultrasonic dispersion of 4 hours, a catalytic amount of DPA was added to the sonicated solution. Then, this mixture was injected into a silicone tube (inner diameter, 1.0 mm) or onto a screw mold. After 2 hours of the lower–cross-linking curing, the fiber-shaped precursors were formed and demolded from the mold, used as the building blocks for the construction of HAFMS-TSAs.

### Fabrication of HAFMS-TSAs

Generally, the freshly prepared fibrous precursor was mechanically stretched to gain 50% strain, while closely winded onto a designed mandrel with target 3D geometry. The directionally winded 3D fiber array with the mandrel was maintained and fully cured for 48 hours. Then, the mandrel was removed, and mechanically robust HAFMS-TSAs were obtained. Diverse 3D helix structures of LC director fields can be gained by adjusting the winding angle for the fibrous building units. Tunable 3D geometric shapes of the used mandrels allow for the fabrication of various HAFMS-TSAs with complex hierarchical 3D shapes (e.g., S-shaped, helical shape, etc.).

### Fabrication of LC order–graded HAFMS-TSAs

To gain graded LC order in a HAFMS-TSA, the varying stretching force was applied to the fibrous precursor during the winding process. When winding the fibrous precursor around the mandrel, the stretching force was gradually increased, which leads to the graded increase of the stretching ratio for the stretched fiber precursor, resulting in the gradually increasing LC order along the longitudinal direction of the winded HAFMS-TSA. By changing the stretching ratio of the fibers during the winding process, we can control local LC order on demand, thus achieving regulation of local deformations (e.g., radial deformations and torsional deformations) at any desired part of a single HAFMS-TSA.

### Programmable materials for a HAFMS-TSA

For programming materials in a single HAFMS-TSA, a fibrous precursor with locally variable material composition must be prepared. For example, to gain the HAFMS-TSA composed of two different materials (Fig. 4F), a fibrous precursor, half of which consisted of NIR-responsive LCE and the other half of which consisted of UV-responsive LCEs must be fabricated. First, the solution of a mixture (1.67:1 molar ratio of RM82/DODT and 3:1 molar ratio of DODT/PETMP; 2 wt% graphene, a catalytic amount of DPA) in chloroform was injected onto a screw mold to fill the grooves of half of the screw mold. Then, the other solution of a mixture (1.67:1 molar ratio of RM82/DODT and 3:1 molar ratio of DODT/PETMP; 5 wt% photoresponsive azobenzene monomer, a catalytic amount of DPA) was injected onto the other half of the screw mold and fill the rest of the grooves. After 2 hours for the lower–cross-linking curing, the single fibrous precursor half composed of NIR-responsive LCE and the other half made of UV-responsive LCE were formed and demolded from the mold, used as the building block for the following construction of two-material programmable HAFMS-TSAs. The winding fabrication for these multiple-material HAFMS-TSAs is similar to that described here for preparing single-material HAFMS-TSAs. We can not only program a wide variety of material compositions in a single fibrous precursor but also locally regulate material composition at any position, which allows us to locally tune materials and stimuli responses for the as-prepared material-programmable HAFMS-TSAs.

### Fluid pumping driven by the wringing motion

The pumping system was composed of a HAFMS-TSA (θ = 67^o^), an elastic tube (Ecoflex), silicone tubes, and two check valves (Fig. 5B). As shown in Fig. 5C, the HAFMS-TSA was connected to the elastic tube; they are working together as the core driving component for the system. The silicon tubes serve as the fluid conduits. The two check values make the fluid flow in one direction. The HAFMS-TSA with θ = 67^o^ closed to θ_2_ was selected for the pumping system to gain the strong wringing motion. Upon light irradiation, the twisting motion of the irradiated HAFMS-TSA can wring the connected elastic tube, squeezing the fluid out of the deformed tube, thus achieving powerful pumping functions.

### Theoretical model

We modeled the HAFMS-TSA using Fung orthotropic material in ABAQUS (*44*), in which 11 material properties in the model were determined by executing genetic algorithm using Mcalibration software (*45*). Table S2 shows the fitted material parameters from Mcalibration software.

An analogy between the light-induced deformation and thermal strain was established. On the basis of the assumption of constant volume, i.e., the initial volume of the fiber $V_{0}=\pi R_{0}^{2}L_{0}$ remains unchanged after the light exposure, *V*′ = π(*R*_0_ + ∆*R*)^2^(*L*_0_ + ∆*L*), where *R*_0_, *L*_0_, ∆*R*, and ∆*L* are the initial radius, the length of fiber, the radius change, and the length change of fiber, respectively. By equating *V*_0_ and *V*′, we obtain

$$\frac{\text{Δ}R}{R_{0}}=\frac{1}{\sqrt{\frac{\text{Δ}L}{L_{0}}+1}}-1$$ (1)

In the simulation, the axial change of the fiber ($\text{Δ}L/L_{0}$) during light exposure was obtained experimentally, while the lateral change ($\text{Δ}R/R_{0}$) was calculated according to the above formula. Figure S13 shows the axial change and lateral change of the fiber as a function of temperature. Note that the temperature here is a dummy variable and only relates to the intensity of light.

The thermal expansion coefficients in ABAQUS were calculated according to the following equations

$$\alpha_{1}=\frac{\text{Δ}L/L_{0}}{T-T_{0}}$$ (2)

$$\alpha_{2}=\alpha_{3}=\frac{\text{Δ}R/R_{0}}{T-T_{0}}$$ (3)

where α_1_ is thermal expansion coefficient of axial deformation, α_2_ and α_3_ are the thermal expansion coefficients of lateral deformation, *T*_0_ is the initial reference temperature, and *T* is the temperature after light exposure. Hence, the orthotropic expansion coefficients were obtained, as shown in table S3. Solid element C3D8R was used in the simulation; all the analysis was performed in ABAQUS/Standard.

## Supplementary Materials

### This PDF file includes:

Supplementary Materials and Methods Figs. S1 to S13 Tables S1 to S3 Legends for movies S1 to S8 References
