# Supplementary material for: Bioinspired helical-artificial fibrous muscle structured tubular soft actuators
Source: Sci Adv. 2023 Jun 23;9(25):eadh3350. doi: 10.1126/sciadv.adh3350 (PMC10289666; doi:10.1126/sciadv.adh3350)
Supplement: Supplementary file 1 — Supplementary Materials and Methods Figs. S1 to S13 Tables S1 to S3 Legends for movies S1 to S8 References [file sciadv.adh3350_sm.pdf]

Science Advances
AAAS

Supplementary Materials for

Bioinspired helical-artificial fibrous muscle structured tubular soft actuators

Zhiming Hu *et al.*

Corresponding author: Jiu-an Lv, lvjiuan@westlake.edu.cn; Hanqing Jiang, jianghanqing@westlake.edu.cn

Sci. Adv. **9**, eadh3350 (2023)
DOI: 10.1126/sciadv.adh3350

The PDF file includes:

Supplementary Materials and Methods
Figs. S1 to S13
Tables S1 to S3
Legends for movies S1 to S8
References

Other Supplementary Material for this manuscript includes the following:

Movies S1 to S8

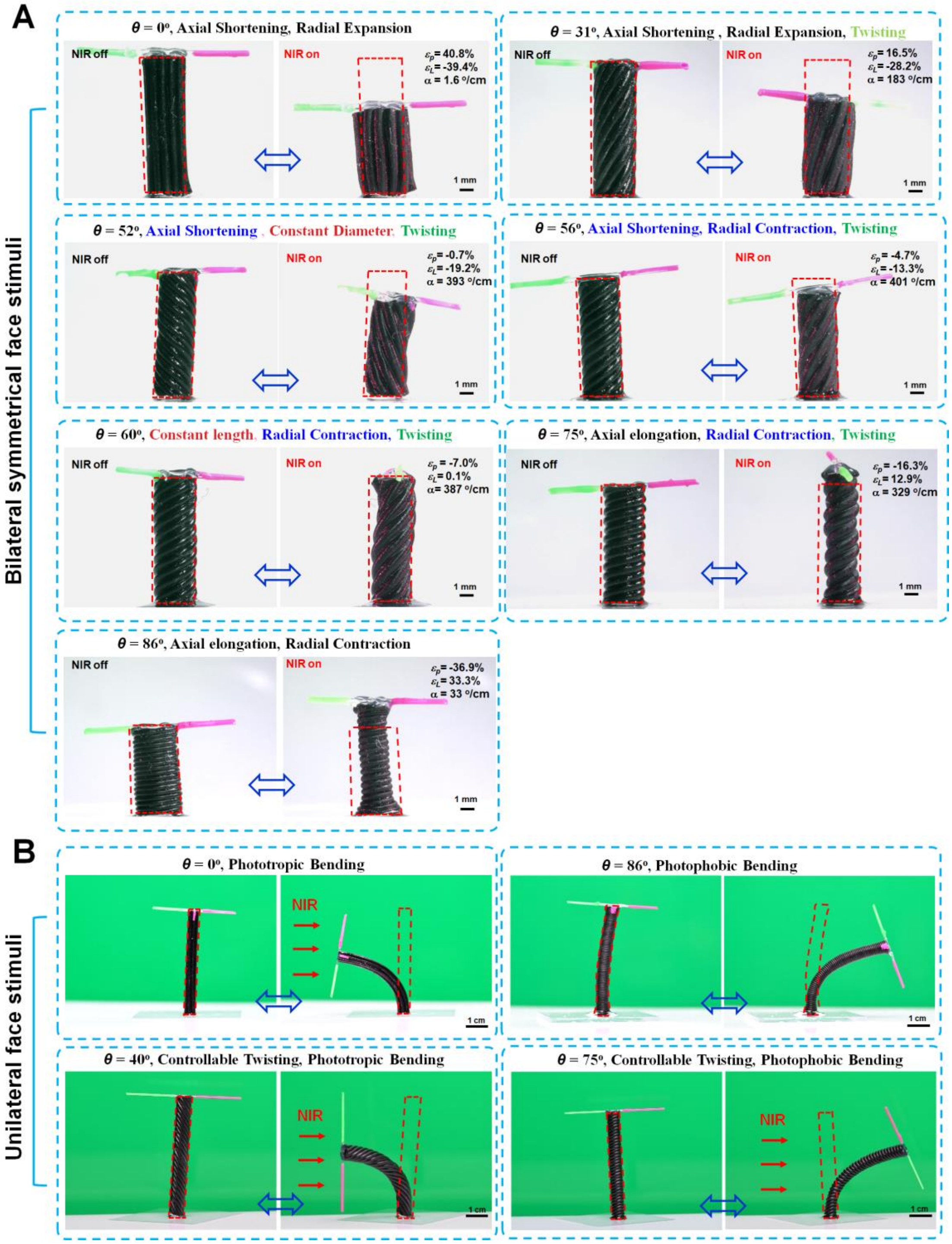

Fig. S1. Diverse morphing modes generated by HAFMS-TSAs. (A) Experimental photographs showing 7 morphing modes of HAFMS-TSAs upon bilateral symmetrical face stimuli. **(B)** Experimental photographs showing 4 morphing modes of HAFMS-TSAs upon unilateral face stimuli. The red arrows indicate the incident direction of the light. The red dashed frames denote the initial shape and size of the HAFMS-TSAs before external stimuli. The colored bar at the top of the HAFMS-TSAs was used to clearly display the twisting motion of the morphing HAFMS-TSAs.

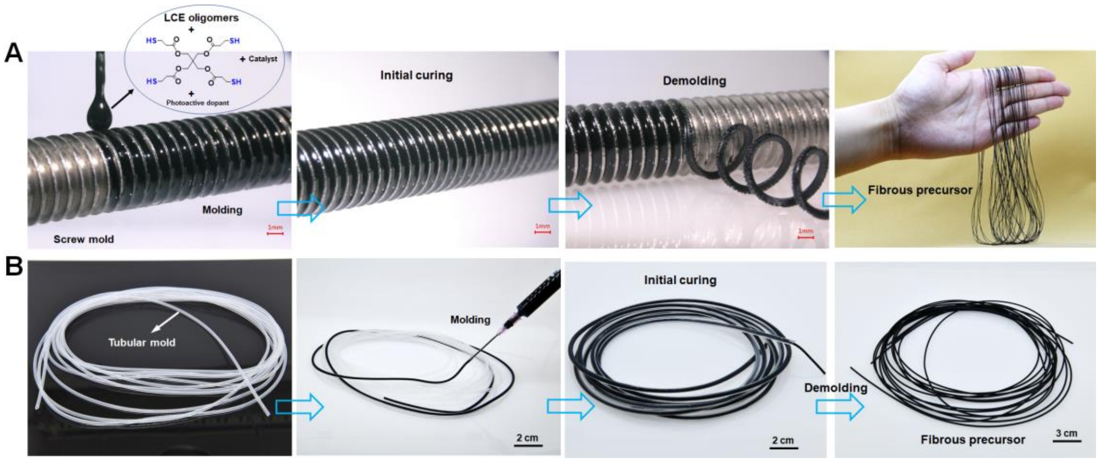

Fig. S2. Preparation of the fibrous precursors for modular fabrication of HAFMS-TSAs. (A) Experimental photographs showing the processing procedures using the screw mold to produce the fibrous precursors. **(B)** Experimental photographs displaying the fabrication procedures using the tubular mold to gain the fibrous precursors.

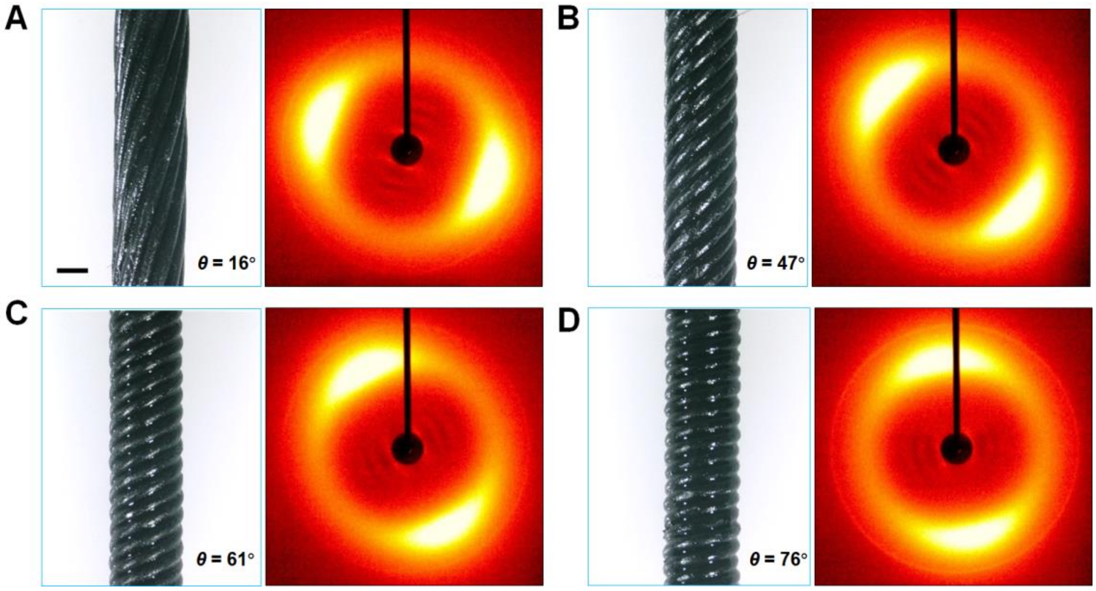

Fig. S3. Tunable 3D helix of LC director fields. Experimental photographs showing the morphologies and 2D-WXRD patterns for HAFMS-TSAs with $\theta = 16°$ (A), 47° (B), 61° (C), and 76° (D), respectively.

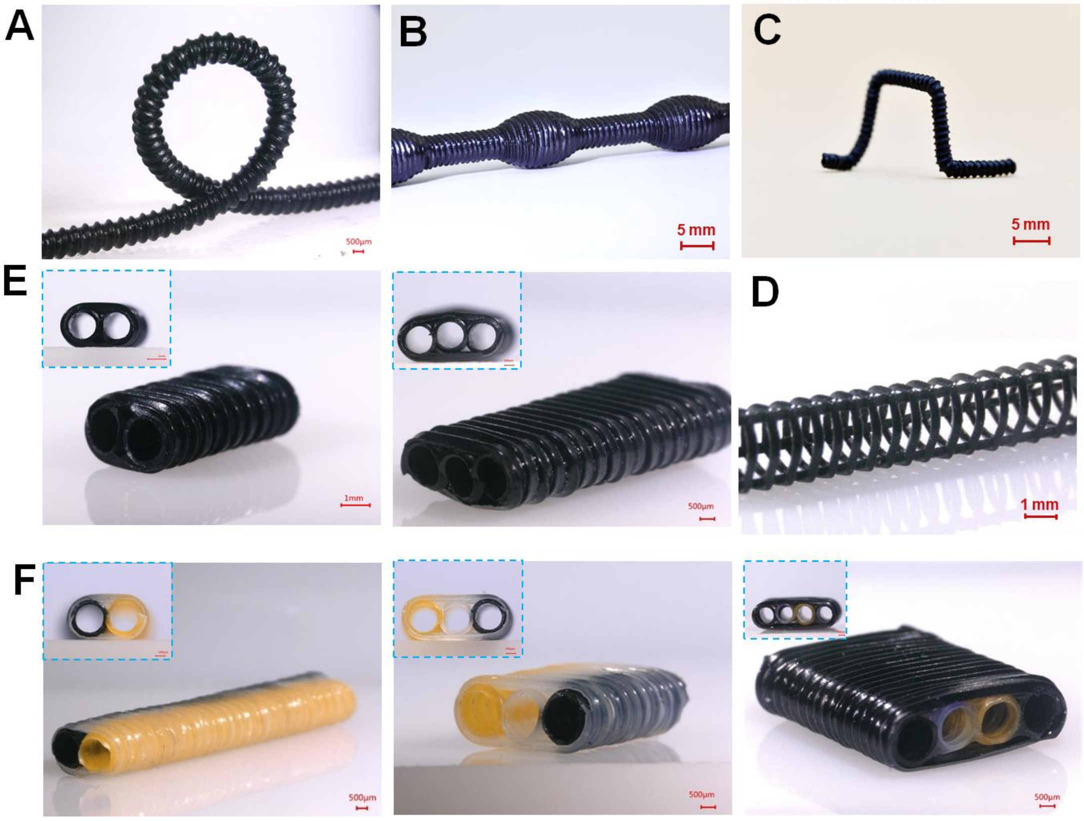

Fig. S4. Tunable 3D geometries of HAFMS-TSAs. (A) A coiled HAFMS-TSA. **(B)** Pearl-chain-shaped HAFMS-TSA. **(C)** A self-standing football-gate-shaped HAFMS-TSA. **(D)** A wall-hollowed-out HAFMS-TSA. **(E)** Multi-level structured HAFMS-TSAs. **(F)** Multi-level, multi-materials structured complex HAFMS-TSAs.

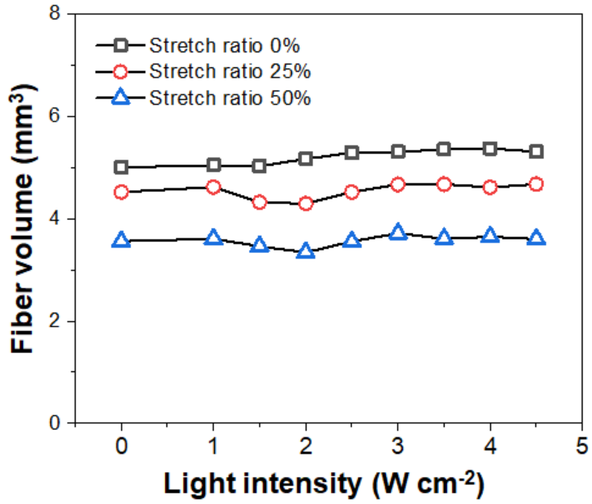

Fig. S5. The volume of the fiber units upon light irradiation with different intensities. The volume of the fibrous building blocks maintains constant when deformed, which means any decrease in one dimension will simultaneously lead to a compensatory increase in at least on one other dimension.

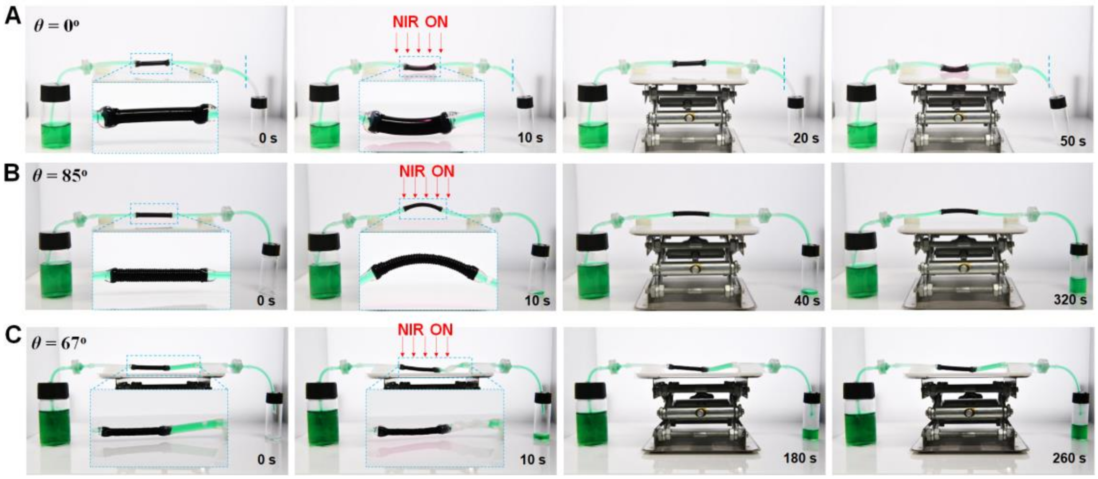

Fig. S6. Active and tunable pumping functions of HAFMS-TSAs. (A) Controlled Experiment showing that tubular LCE with simple orientation along their long axis fails in pumping fluids via its photodeformation. **(B)** Experimental photographs showing fluid pumping driven by leveraging the light-driven cavity volume change of a HAFMS-TSA. **(C)** Large ejection fraction achieved by wringing motion, which is induced by the twisting motion of a HAFMS-TSA. The intensity of the NIR light is ~ 2.5 W cm^{-2}, the length and the inner diameter of the HAFMS-TSA are 3 cm and 3 mm, respectively. Periodic light irradiation (cycles of light-on 10 s and light-off 10 s) was used to drive the pumping of the HAFMS-TSA.

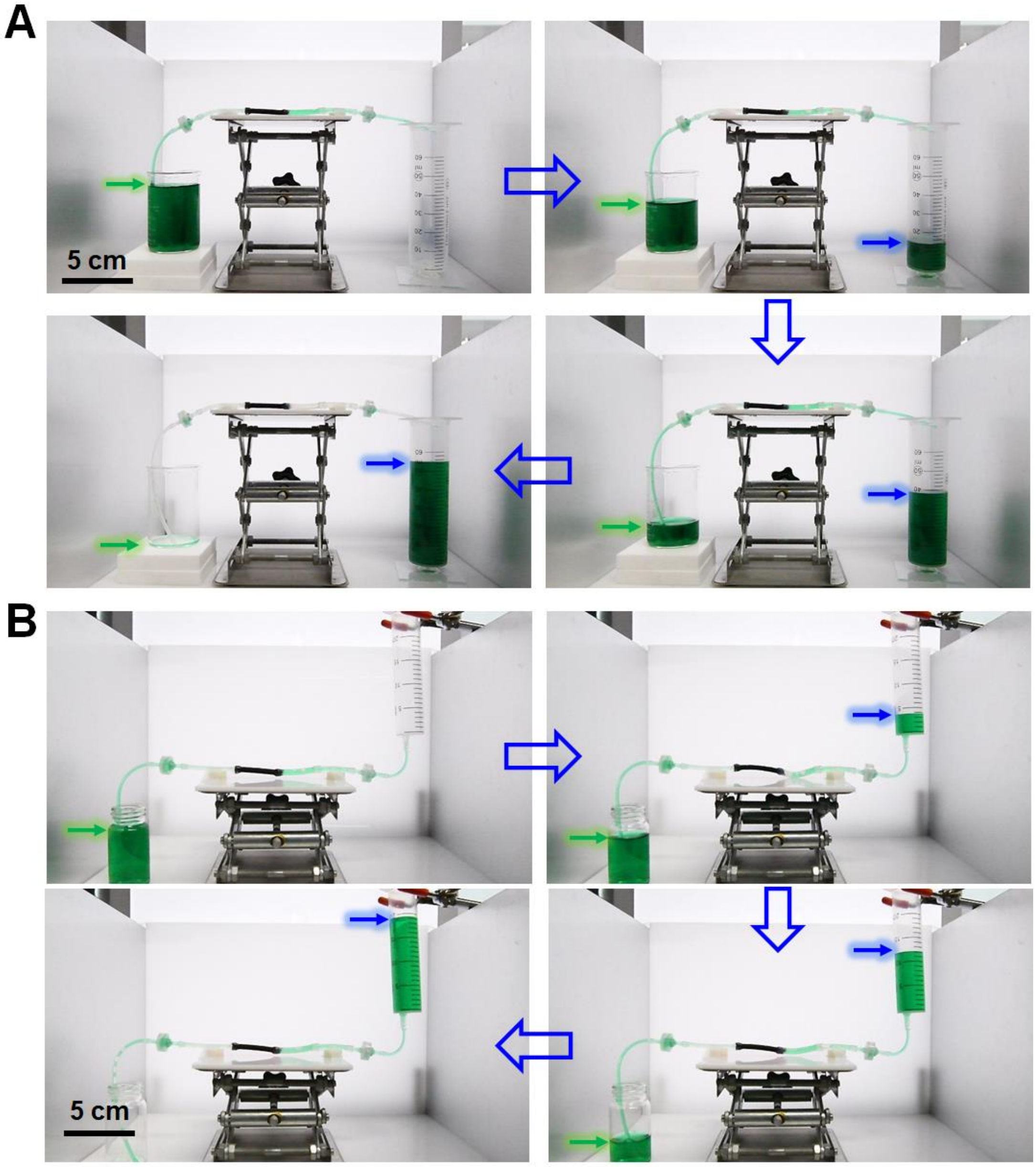

Fig. S7. Powerful Pumping functionality of HAFMS-TSAs. (A) Experimental photographs showing fluid pumping driven by the photodeformation of the HAFMS-TSA**. (B)** Wringing motion enables the system to generate powerful force to overcome gravity to pump and lift fluid upward. Periodic NIR light generated by light on 10 s and off 10 s alternatively was employed to drive the pumping of the system. The intensity of the NIR light is ~ 2.5 W cm^{-2}, the inner diameter of the HAFMS-TSAs is 3 mm.

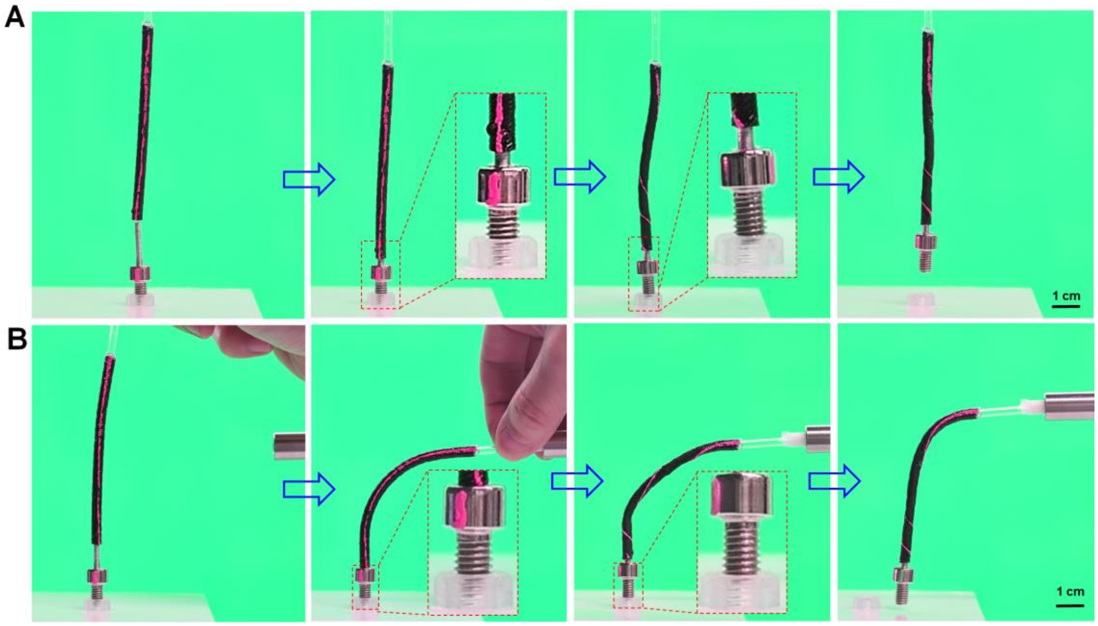

Fig. S8. HAFMS-TSA serves as a small-scale soft-robotic tentacle. (A) Experimental photographs showing a HAFMS-TSA grabbing a bolt through photo-driven radical contraction and unscrewing it via the photo-driven twisting motion. **(B)** Experimental photographs showing a HAFMS-TSA grabbing and exerting torque to a bolt even in its curved state. The red marker on the wall of the HAFMS-TSA is used to indicate the twisting motions in its straight state and the curved state upon light irradiation, respectively. The intensity of the NIR light is ~ 2 W cm^{-2}.

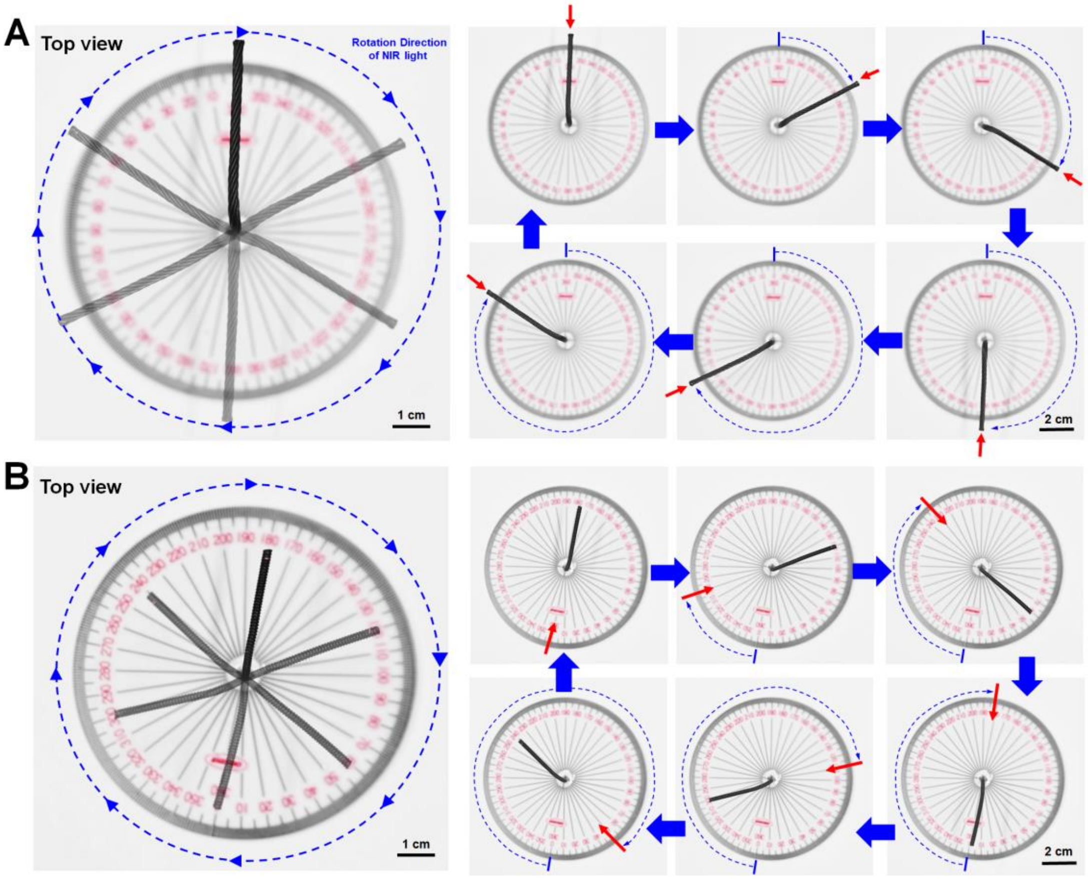

Fig. S9. Reorientation motion of a HAFMS-TSA in response to directional light with varying azimuth angles. (A) Top-view snapshots showing the HAFMS-TSA (θ=21°) continuously, autonomously phototropic reorientating to the direction of the incident light with varying azimuth angles. **(B)** Top-view snapshots exhibiting adaptive photophobic reorientation of the HAFMS-TSA (θ=83°) driven by directional light with varying azimuth angles. The intensity of the NIR light is ~ 1.5 W cm^{-2}, The inner diameter of the HAFMS-TSA is 1 mm.

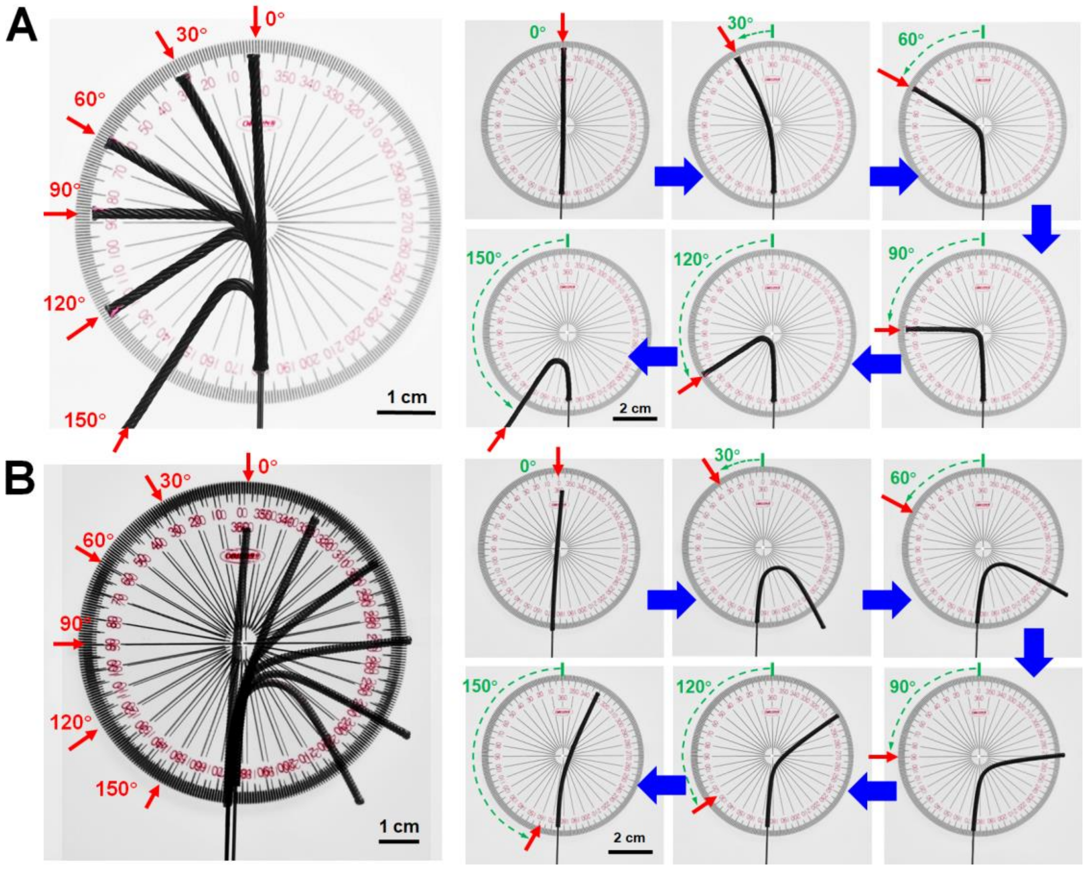

Fig. S10. Reorientation motion of a HAFMS-TSA in response to directional light with varying zenithal angles. (A) Adaptive phototropic reorientation of the HAFMS-TSA (θ=21°) upon the light irradiation with varying zenithal angles. **(B)** Adaptive photophobic reorientation of the HAFMS-TSA (θ=83°) upon light irradiations with varying zenithal angles. The intensity of the NIR light is ~ 1.5 W cm^{-2}, the inner diameter of the HAFMS-TSAs is 1 mm.

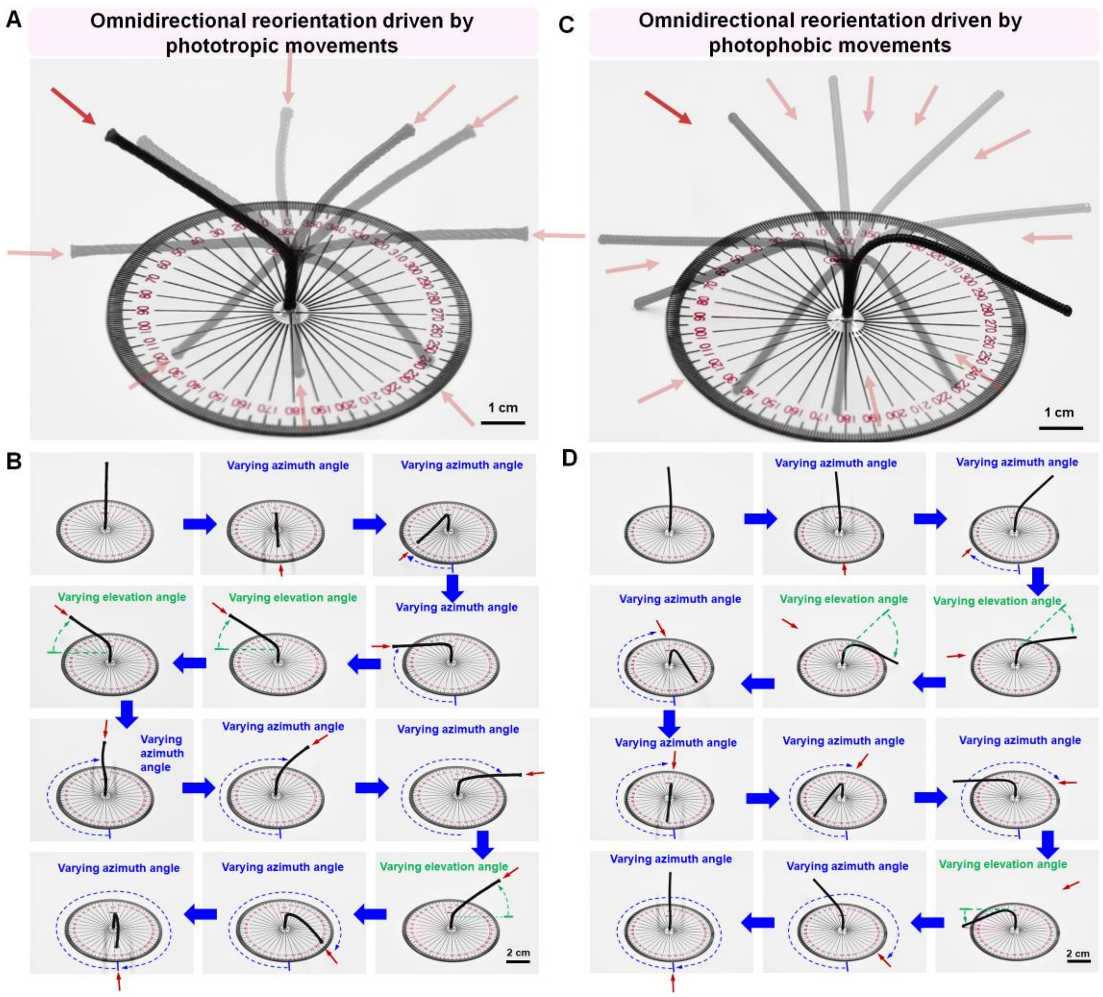

Fig. S11. Omnidirectional reorientations driven by phototropic movements or photophobic movements. (A) Adaptive phototropism and omnidirectional light-tracking performance of phototropic HAFMS-TSA with θ =21°. **(B)** Lateral-view snapshots exhibiting a HAFMS-TSA (θ = 21°) continuously tracking the light that revolves azimuthally or zenithally around the HAFMS-TSA. **(C)** Adaptive and omnidirectional away-from-light reorientations of HAFMS-TSA with θ =83°. **(D)** Lateral-view snapshots exhibiting a HAFMS-TSA (θ = 83°) continuously reorientating away from the light that revolves azimuthally or zenithally around the HAFMS-TSA. The red arrows denote the incident directions of the light. The dashed blue lines and the dashed green lines indicate the change of the azimuth angles and the change of the zenith angles of the incident light, respectively. The inner diameter of the HAFMS-TSAs is 1 mm. The intensity of the NIR light is ~ 1.5 W cm^{-2}.

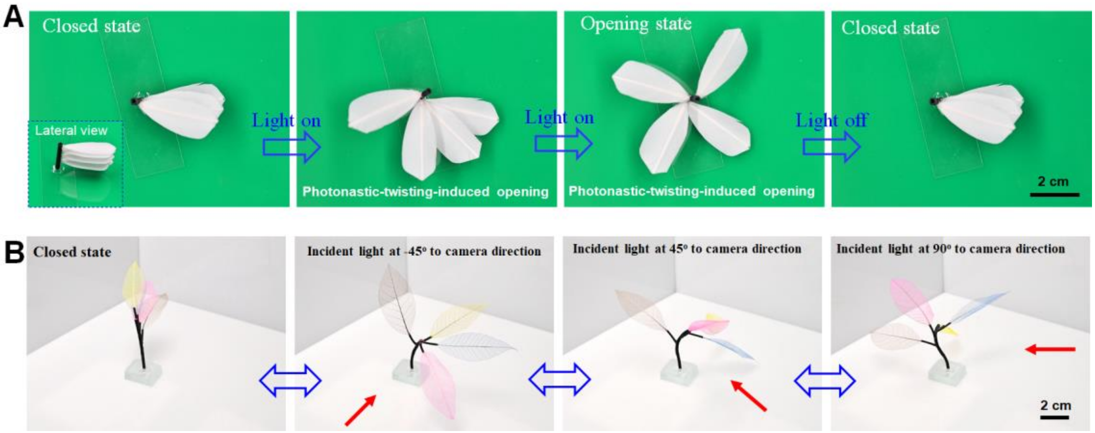

Fig. S12. Adaptive and automatic shape transformations of intelligent artificial plants (ILPAs) in response to environmental stimuli. (A) Opening behavior induced by photonastic twisting motion. **(B)** Directional opening of IPA upon light irradiation with varied incident direction. The red arrow indicates the incident direction of the light. The light intensity is ~ 2 W cm^{-2}.

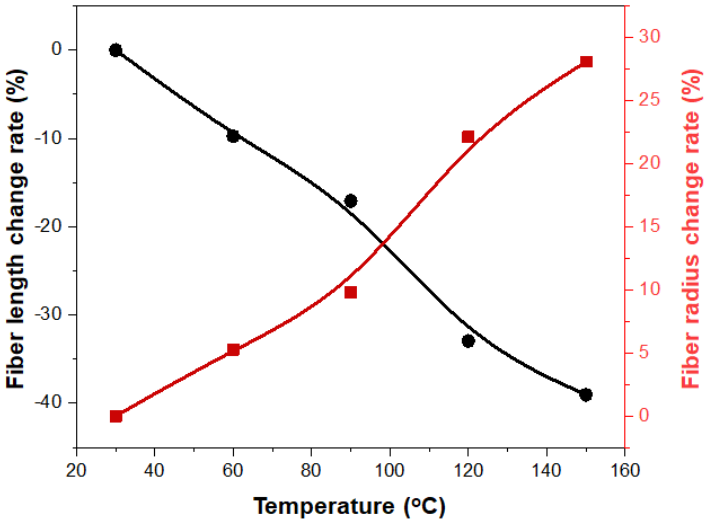

Fig. S13. The axial and radius change rate of the fiber as function of temperature. The temperature in here is a dummy variable, and only relates to the intensity of light.

Table S1. The summary of deformation modes and active soft materials for reported tubular soft actuators.

Active soft materials	Stimuli	Deformation modes	Ref.
Hydrogels	Heat	Bending, Twisting, Shrinkage	[4]
Hydrogel	Heat	Contraction	[46]
Liquid crystal polymer	Light	Contraction	[1]
Liquid Crystal Polymers	Light	Contraction	[47]
Liquid crystal polymers	Light	Contraction	[48]
Liquid crystal polymer	Heat	Contraction	[19]
Polyethylene	Heat	Twisting	[31]
Liquid Crystal Elastomer	Hot water	Contraction	[49]
Liquid Crystal Elastomer	Heat	Contraction	[50]
Liquid Crystal Elastomer	Light	Bending	[15]
Liquid Crystal Elastomer	Light	Contraction， Bending	[20]
Liquid Crystal Elastomer	Electrical	Contraction, Bending	[14]
Liquid Crystal Elastomer	Heat or light	Contraction, Bending	[38]
Liquid Crystal Elastomer	Heat	Contraction	[51]
Liquid Crystal Elastomer	Light	Twisting, Shortening, Elongation, Expansion, Contraction, Phototropic Bending, Photophobic Bending	**This work**

Table S2. Fitted Fung-Orthotropic material parameters

Fung-Orthotropic material parameters	
b1111	0.1805
b1122	-0.1496
b2222	0.1552
b1133	-0.0664
b2233	0.0430
b3333	0.0567
b1212	1
b1313	1
b2323	1
C (N/mm^2)	128.115
D (mm^2/N)	0.01

Table S3. The orthotropic thermal expansion coefficients

Temperature	Thermal expansion coefficients		
	α_1 (1/^{o}C)	α_2 (1/^{o}C)	α_3 (1/^{o}C)
30	-0.00325333	0.00175623	0.00175623
60	-0.00325333	0.00175623	0.00175623
90	-0.00284667	0.0016362	0.0016362
120	-0.00366	0.0024572	0.0024572
150	-0.00325333	0.00233991	0.00233991

Supplementary movie descriptions

Movie S1. Seven morphing modes of HAFMS-TSAs upon bilateral symmetrical face stimuli.

Mode 1: Fiber angle 0°, Axial Shortening & Radial Expansion.

Mode 2: Fiber angle 31°, Axial Shortening & Radial Expansion & Twisting.

Mode 3: Fiber angle 52°, Axial Shortening & Constant Diameter & Twisting.

Mode 4: Fiber angle 56°, Axial Shortening & Radial Contraction & Twisting.

Mode 5: Fiber angle 60°, Constant length & Radial Contraction & Twisting.

Mode 6: Fiber angle 75°, Axial elongation & Radial Contraction & Twisting.

Mode 7: Fiber angle 86°, Axial elongation & Radial Contraction.

The intensity of the NIR light is ~ 1.5 W cm^{-2}, and the inner diameter of the HAFMS-TSAs is 2 mm.

Movie S2. Four morphing modes of HAFMS-TSAs upon unilateral face stimuli.

Mode 8: Fiber angle 0°, Phototropic Bending. The inner diameter of the tubular actuator is 1 mm.

Mode 9: Fiber angle 40°, Controllable Twisting & Phototropic Bending. The inner diameter of the tubular actuator is 2 mm.

Mode 10: Fiber angle 75°, Controllable Twisting & Photophobic Bending. The inner diameter of the tubular actuator is 2 mm.

Mode 11: Fiber angle 86°, Photophobic Bending. The inner diameter of the tubular actuator is 2 mm.

The intensity of the NIR light is ~ 1.5 W cm^{-2}.

Movie S3. Local tunable LC order, materials, and actuations in a single HAFMS-TSA.

Part 1: Graded LC order enables a HAFMS-TSA to transform from a symmetric (cylindrical) shape into an asymmetric (tapered) shape when exposed to light irradiation.

Part 2: Locally-varied LC orders allow the generation of five regions with different shape changes in a single HAFMS-TSA.

Part 3: A material-programmed HAFMS-TSA made of NIR-responsive material and UV-responsive material shows locally-tunable photodeformation. Under the irradiation of either NIR or UV light, the local change in the diameter of the composited actuator can be induced, while the overall change of the diameter can be generated upon the simultaneous irradiation of NIR and UV light.

Part 4: A material-programmed HAFMS-TSA composed of alternating non-photoresponsive and photoresponsive LCE materials reversibly transformed between a cylinder structure and a pearl-necklace-liked structure by light.

The intensity of the NIR light is ~ 1.5 W cm^{-2}. The intensity of the UV light is ~ 550 mW cm^{-2}. The inner diameter of the HAFMS-TSA is 4 mm.

Part 5: Locally tune the actuation strain of a HAFMS-TSA to effectively avoid stress-concentration-induced detaching. HAFMS-TSA without changing local actuation strain/stress, glued on a glass substrate, detaching from a glass substrate upon light irradiation whereas the HAFMS-TSA with locally-reduced actuation strain/stress still stayed attached to the substrate. The intensity of the NIR light is ~ 2.5 W cm^{-2}. The mass of the steel ball is 5 g.

Movie S4. Fluid pumping driven by leveraging the cavity volume change of HAFMS-TSA upon light irradiation.

Fluid pumping driven by leveraging the cavity volume change of HAFMS-TSA upon alternating NIR light irradiation (NIR on: 10 s, NIR off: 10 s). The intensity of the NIR light is ~ 2.5 W cm^{-2}. The inner diameter and length of the tubular actuator are ~3 mm and ~3 cm, respectively.

Movie S5. Fluid pumping driven by the twisting motion of the HAFMS-TSA.

The wringing deformation driven by the twisting motion of the HAFMS-TSA, and it causes torsional buckling of the elastic tube, leading to its collapse and squeezing the most fluid out of the wrung tube. The length and the inner diameter of the HAFMS-TSA are is 3 cm and 3 mm, respectively.

Part 1: Alternating NIR light (NIR on 25 s, NIR off: 25 s). The intensity of the NIR light is ~ 2.0 W cm^{-2}.

Part 2: Alternating NIR light (NIR on 10 s, NIR off: 10 s). The intensity of the NIR light is ~ 2.5 W cm^{-2}.

Movie S6. HAFMS-TSA serve as a small-scale soft-robotic tentacle.

Part 1: HAFMS-TSA grabs a bolt through photo-driven radical contraction and unscrew it in a straight state.

Part 2: Even in curved states, HAFMS-TSA can exert torque on a bolt.

Part 3: HAFMS-TSA adapt to a meandering pipe to unscrew a bolt through photo-driven twisting motion.

The intensity of the NIR light is ~ 2.0 W cm^{-2}. The inner diameter and length of the tubular actuator are ~3 mm and ~8 cm, respectively. The mass of the screw is 5.2 g.

Movie S7. Omnidirectional reorientation driven by phototropic movements or photophobic

movements.

Part 1: Phototropic movements *vs* photophobic movements.

Part 2: Phototropic movements and photophobic movements when the incident direction of the light was maintained but the HAFMS-TSA was continuously rotating.

Part 3: Photonastic-twisting-driven opening behaviors.

The intensity of the NIR light is ~ 1.5 W cm^{-2}, The inner diameter of the HAFMS-TSAs is 1 mm.

Movie S8. Intelligent artificial plants constructed by HAFMS-TSAs with varied light-reorientation movements.

The lower part (length 15 mm, inner diameter 2 mm) is made of the HAFMS-TSA with $\theta = 85°$ to enable photophobic movements, and the upper part (length 40 mm, inner diameter 2 mm) consists of tubular LCE with $\theta = 40°$ to produce phototropic movements and large photonastic twisting. The branches are made of the HAFMS-TSA with a large θ of 85°. The intensity of the NIR light is ~ 2 W cm^{-2}.

REFERENCES AND NOTES

1. J.-A. Lv, Y. Liu, J. Wei, E. Chen, L. Qin, Y. Yu, Photocontrol of fluid slugs in liquid crystal polymer microactuators. *Nature* **537**, 179–184 (2016).

2. X.Wen, S. Sun, P. Wu, Dynamic wrinkling of a hydrogel–elastomer hybrid microtube enables blood vessel-like hydraulic pressure sensing and flow regulation. *Mater. Horiz.* **7**, 2150–2157 (2020).

3. W. Lei, G. Hou, M. Liu, Q. Rong, Y. Tian, L. Jiang, High-speed transport of liquid droplets in magnetic tubular microactuators. *Sci. Adv.* **4**, eaau8767 (2018).

4. H. Lin, S. Ma, B. Yu, M. Cai, Z. Zheng, F. Zhou, W. Liu, Fabrication of asymmetric tubular hydrogels through polymerization-assisted welding for thermal flow actuated artificial muscles. *Chem. Mater.* **31**, 4469–4478 (2019).

5. T. Zhao, W. Fang, Y. Fan, Z. Hu, H. Wu, X. Feng, J-A. Lv, Phototactic miniature soft robots with terrain adaptability. *Adv. Mater. Technol.* **7**, 2101660 (2022).

6. Q. He, Z. Wang, Y. Wang, Z. Wang, C. Li, R. Annapooranan, J. Zeng, R. Chen, S. Cai, Electrospun liquid crystal elastomer microfiber actuator. *Sci. Robot.* **6**, eabi9704 (2021).

7. B. Gupta, L. Zhang, A. A. Melvin, B. Goudeau, L. Bouffier, A. Kuhn, Designing tubular conducting polymer actuators for wireless electropumping. *Chem. Sci.* **12**, 2071–2077 (2021).

8. S. Gantenbein, K. Masania, W. Woigk, J. P. W. Sesseg, T. A. Tervoort, A. Studart, Three-dimensional printing of hierarchical liquid-crystal-polymer structures. *Nature* **561**, 226–230 (2018).

9. Y. Guo, J. Zhang, W. Hu, M. T. A. Khan, M. Sitti, Shape-programmable liquid crystal elastomer structures with arbitrary three-dimensional director fields and geometries. *Nat. Commun.* **12**, 5936 (2021).

10. K. M. Herbert, H. E. Fowler, J. M. McCracken, K. R. Schlafmann, J. A. Koch, T. J. White, Synthesis and alignment of liquid crystalline elastomers. *Nat. Rev. Mater.* **7**, 23–38 (2022).

11. A. Kotikian, J. M. Morales, A. Lu, J. Mueller, Z. S. Davidson, J. W. Boley, J. A. Lewis, Innervated, self-sensing liquid crystal elastomer actuators with closed loop control. *Adv. Mater.* **33**, 2101814 (2021).

12. S. Li, M. M. Lerch, J. T. Waters, B.Deng, R. S. Martens, Y. Yao, D. Y.Kim, K. Bertoldi, A. Grinthal, A. C. Balazs, J. Aizenberg, Self-regulated non-reciprocal motions in single-material microstructures. *Nature* **605**, 76–83 (2022).

13. Y. Wang, J. Liu, S. Yang, Multi-functional liquid crystal elastomer composites. *Appl. Phys. Rev.* **9**, 011301 (2022).

14. Q. He, Z. Wang, Y. Wang, A. Minori, M. T. Tolley, S. Cai, Electrically controlled liquid crystal elastomer–based soft tubular actuator with multimodal actuation. *Sci. Adv.* **5**, eaax5746 (2019).

15. H. Liu, H. Tian, X. Li, X. Chen, K. Zhang, H. Shi, C. Wang, J. Shao, Shape-programmable, deformation-locking, and self-sensing artificial muscle based on liquid crystal elastomer and low–melting point alloy. *Sci. Adv.* **8**, eabn5722 (2022).

16. W. M. Kier, K. K. Smith, Tongues, tentacles and trunks: The biomechanics of movement in muscular-hydrostats. *Zool. J. Linn. Soc.* **83**, 307–324 (1985).

17. J. Zou, M. Feng, N. Ding, P. Yan, H. Xu, D. Yang, N. X Fang, G. Gu, X. Zhu, Muscle-fiber array inspired, multiple-mode, pneumatic artificial muscles through planar design and one-step rolling fabrication. *Natl. Sci. Rev.* **8**, nwab048 (2021).

18. D. Trivedi, C. D. Rahn, W. M. Kier, I. D. Walker, Soft robotics: Biological inspiration, state of the art, and future research. *Appl. Bionics Biomech.* **5**, 99–117 (2008).

19. L. Yu, R. Peng, G. Rivers, C. Zhang, P. Si, B. Zhao, Multifunctional liquid crystal polymer network soft actuators. *J. Mater. Chem. A* **8**, 3390–3396 (2020)**.**

20. X. Qian, Q. Chen, Y. Yang, Y. Xu, Z. Li, Z. Wang, Y. Wu, Y. Wei, Y. Ji, Untethered recyclable tubular actuators with versatile locomotion for soft continuum robots, *Adv. Mater.* **30**, 1801103 (2018).

21. H. Rodrigue, W. Wei, B. Bhandari, S.-H. Ahn, Fabrication of wrist-like SMA-based actuator by double smart soft composite casting. *Smart Mater. Struct.* **24**, 125003 (2015).

22. G. Singh, G. Krishnan, An isoperimetric formulation to predict deformation behavior of pneumatic fiber reinforced elastomeric actuators, *2015 IEEE/RSJ International Conference on Intelligent Robots and Systems (IROS)* (IEEE, 2015), pp. 1738–1743.

23. F. Connolly, C. J. Walsh, K. Bertoldi, Automatic design of fiber-reinforced soft actuators for trajectory matching. *Proc. Natl. Acad. Sci. U.S.A.* **114**, 51–56 (2016).

24. F. Connolly, P. Polygerinos, C. J. Walsh, K. Bertoldi, Mechanical programming of soft actuators by varying fiber angle. *Soft Robot.* **2**, 26–32 (2015).

25. J. Yan, X. Zhang, B. Xu, J. Zhao, A new spiral-type inflatable pure torsional soft actuator. *Soft Robot.* **5**, 527–540 (2018).

26. M. Schaffner, J. A. Faber, L. Pianegonda, P. A. Rühs, F. C. A. R. Studart, 3D printing of robotic soft actuators with programmable bioinspired architectures. *Nat. Commun.* **9**, 878 (2018).

27. G. Singh, G. Krishnan, A constrained maximization formulation to analyze deformation of fiber reinforced elastomeric actuators. *Smart Mater. Struct.* **26**, 065024 (2017).

28. R. V. Martinez, C. R. Fish, X. Chen, G. M. Whitesides, Elastomeric origami: Programmable paper-elastomer composites as pneumatic actuators. *Adv. Funct. Mater.* **22**, 1376–1384 (2012).

29. B. Gorissen, T. Chishiro, S. Shimomur, D. Reynaerts, M. D. Volder, S. Konishi, Flexible pneumatic twisting actuators and their application to tilting micromirrors. *Sens. Actuators A* **216**, 426–431 (2014).

30. J. Choi, S. H. Ahn, K.-J. Cho, Design of fully soft actuator with double-helix tendon routing path for twisting motion, *2020 IEEE/RSJ International Conference on Intelligent Robots and Systems (IROS)* (IEEE, 2020), pp. 661–8666.

31. S. Li, R. Zhang, G. Zhang, L. Shuai, W. Chang, X. Hu, M. Zou, X. Zhou, B. An, D. Qian, Z. Liu, Microfluidic manipulation by spiral hollow-fibre actuators. *Nat. Commun.* **13**, 1331 (2022).

32. X. Xia, C. M. Spadaccini, J. R. Greer, Responsive materials architected in space and time. *Nat. Rev. Mater.* **7**, 683–701 (2022).

33. X. Ni, H. Luan, J.-T. Kim, S. I. Rogge, Y. Bai, J. W. Kwak, S. Liu, D. S. Yang, S. Li, S. Li, Z. Li, Y. Zhang, C. Wu, X. Ni, Y. Huang, H. Wang, J. A. Rogers, Soft shape-programmable surfaces by fast electromagnetic actuation of liquid metal networks. *Nat. Commun.* **13**, 5576 (2022).

34. H. Wang, R. Zhang, W. Chen, X. Wang, R. Pfeifer, A cable-driven soft robot surgical system for cardiothoracic endoscopic surgery: Preclinical tests in animals. *Surg. Endosc.* **31**, 3152–3158 (2017).

35. R. V. Martinez, J. L. Branch, C. R. Fish, L. Jin, R. F. Shepherd, R. M. D. Nunes, Z. Suo, G. M. Whitesides, Robotic tentacles with three-dimensional mobility based on flexible elastomers. *Adv. Mater.* **25,** 205–212 (2013).

36. M. T. Tolley, R. F. Shepherd, B. Mosadegh, K. C. Galloway, M. Wehner, M. Karpelson, R. J. Wood, G. M. Whitesides, A resilient, untethered soft robot. *Soft Robot.* **3**, 213–223 (2014).

37. R. W. Hannan, I. D. Walker, Kinematics and the implementation of an elephant’s trunk manipulator and other continuum style robots. *J. Robot. Syst.* **20**, 45–63 (2003).

38. M. Yang, Y.Xu, X. Zhang, H. K. Bisoyi, P. Xue, Y. Yang, X. Yang, C. Valenzuela, Y. Chen, L.Wang, W. Feng, Q. Li, Bioinspired phototropic MXene-reinforced soft tubular actuators for omnidirectional light-tracking and adaptive photovoltaics. *Adv. Funct. Mater.* **32**, 2201884 (2022).

39. G. D. Buckberg, N. C. Nanda, C. Nguyen, M. J. Kocica, What is the heart? Anatomy, function, pathophysiology, and misconceptions. *J. Cardiovasc. Dev. Dis.* **5**, 33 (2018).

40. E. A. Sallin, Fiber orientation and ejection fraction in the human left ventricle. *Biophys. J.* **9**, 954–964 (1969).

41. X. Chen, X. Zhang, Y. Huang, L. Cao, J. A Liu, A review of soft manipulator research, applications, and opportunities. *J. Field Robot.* **39**, 281–311 (2022).

42. X. Qian, Y. Zhao, Y. Alsaid, X. Wang, M. Hua, T. Galy, H. Gopalakrishna, Y. Yang, J. Cui, N. Liu, M. Marszewski, L. Pilon, H. Jiang, X. He, Artificial phototropism for omnidirectional tracking and harvesting of light. *Nat. Nanotechnol.* **14**, 1048–1055 (2019).

43. J. Ehleringer, I. Forseth, Solar tracking by plants. *Science* **210**, 1094–1098 (1980).

44. ABAQUS analysis user's manual. Version 2018. Dassault Systemes Simulia Corp.

45. Mcalibration. Version 6.7.1, https://polymerfem.com/mcalibration/.

46. H. Lin, S. Ma, B. Yu, M. Cai, Z. Zheng, F. Zhou, W. Liu, Fabrication of asymmetric tubular hydrogels through polymerization-assisted welding for thermal flow actuated artificial muscles. *Chem. Mater.* **31**, 4469–4478 (2019).

47. H. Liu, Y. Zhang, S. Ma, Y. Alsaid, X. Pei, M. Cai, X. He, F. Zhou, Esophagus-inspired actuator for solid transportation via the synergy of lubrication and contractile deformation. *Adv. Sci.* **8**, 2102 (2021).

48. Q. Liu, Y. Liu, J.-A. Lv, E. Chen, Y. Yu, Photocontrolled liquid transportation in microtubes by manipulating mesogen orientations in liquid crystal polymers. *Adv. Intell. Syst.* **1**, 19000 (2019).

49. B. Xu, C. Zhu, L. Qin, J. Wei, Y. Yu, Light-directed liquid manipulation in flexible bilayer microtubes. *Small* **15**, 1901847 (2019).

50. Q. He, Z. Wang, Y. Wang, Z. Song, S. Cai, Recyclable and self-repairable fluid-driven liquid crystal elastomer actuator. *ACS Appl. Mater. Interfaces* **12**, 35464–35474 (2020).

51. X. Liu, S. Kim, X. Wang, Thermomechanical liquid crystalline elastomer capillaries with biomimetic peristaltic crawling function. *J. Mater. Chem. B* **4**, 7293–7302 (2016).

52. B. Ni, G. Liu, M. Zhang, M. Tatoulian, P. Keller, M. Li, Customizable sophisticated three-dimensional shape changes of large-size liquid crystal elastomer actuators. *ACS Appl. Mater. Interfaces* **13**, 54439–54446 (2021).
